# Supplementary material for: Illustrating phylogenetic placement of fossils using RoguePlots: An example from ichneumonid parasitoid wasps (Hymenoptera, Ichneumonidae) and an extensive morphological matrix
Source: PLoS One. 2019 Apr 2;14(4):e0212942. doi: 10.1371/journal.pone.0212942 (PMC6445432; doi:10.1371/journal.pone.0212942)
Supplement: S2 File — Illustrated list of the morphological characters and their states used in this study. (DOCX) [file pone.0212942.s003.docx]

Description of morphological characters

Annotations and abbreviations:

* – see figure in Gauld et al. 2002;

^S^ – supplementary figure at the end of the file;

T1 – tergite one, T2 – tergite two etc;

S1 – sternite one;

[ordered] – character treated as order in the phylogenetic analysis

#1. **Dimensions of mandible** [ordered]

(0) moderately large, weakly and evenly tapered so apex is more than 0.7x base;

(1) moderately large, but more strongly tapered so apex is about 0.6–0.5x base ;

(2) slender and very strongly tapered so apex is <0.4x width at base (Fig. 26, 32*).

#2. **Shape of mandible**

(0) bi-dentate, both tips slightly pointed to evenly rounded (Fig. 1^S^, Fig. 24*);

(1) bi-dentate, one of the tips abruptly broken (chisel-shaped) (Fig. 2^S^);

(2) unidentate, tooth abruptly broken (chisel-shaped);

(3) tridentate (upper tooth subdevided);

(4) unidentate, strongly shortened so that when closed, the mandibles barely overlap or not at all (cf. some species of *Orthocentrus*);

(5) weakly sclerotized and reduced to flap (cf. *Hybrizon*).

Note: State (2) includes twisted mandibles, when second tooth is present on the inside but not visible (cf. Orthocentinae);

#3. **Mandibular teeth length** [ordered]

(0) upper tooth conspicuously the longer, lower tooth small, <0.5 of length of the upper tooth;

(1) upper tooth subequal to the lower (Figs 27, 28*);

(2) upper tooth distinctly shorter than the lower tooth, about 0.6– 0.7 of its length (Fig. 1^S^, Fig. 23*);

(3) upper tooth very small, <0.4 length of lower.

#4. **Mandibular teeth breadth** [ordered]

(0) upper tooth more slender than the lower tooth;

(1) upper tooth similar to or very slightly broader than the lower;

(2) upper tooth conspicuously broader than the lower tooth (Fig. 24*).

#5 **Axis of mandible** [ordered]

(0) not twisted (Figs 27, 28*);

(1) weakly twisted 20–40° (Figs 26, 42*);

(2) twisted about 90° so the inner tooth is not visible when mandibles closed (Fig. 32*).

#6 **Dorsal surface of mandible**

(0) with a cluster of stout long setae in a pit (Fig. 25*);

(1) with sparse scattered pubescence, sometimes glabrous (Fig. 26*);

(2) with a diagonal line of hairs starting where the pit usually is and going down and towards the tip (e.g., *Ophion*).

#7 **Outer surface of mandible**

(0) more or less punctate or slightly rugulose;

(1) strongly striate (Fig. 24*);

(2) with a weakly swollen, polished subapical region;

(3) all smooth;

(4) with swollen, shagreened subapical region.

#8 **Malar space**

(0) without a subocular sulcus or with incomplete sculptured band;

(1) with a sculptured band on the place where sulcus would be, band is complete (extending whole the way through the malar space) (Fig. 3^S^);

(2) with a deep subocular sulcus (Fig. 1^S^).

#9. **Malar space length** [ordered]

(0) obliterated, lower margin of eye more or less touching base of mandible (Fig. 30*);

(1) short to moderately long, 0.2– 0.8x basal mandibular width (Figs 27–29*);

(2) long, 1–1.5x basal mandibular width (Fig. 45*);

(3) very long, >1.8x basal mandibular width.

#10. **Maxillary palp** [ordered]

(0) with five segments;

(1) with four segments;

(2) with three segments.

#11. **Labial palp**

(0) with four segments;

(1) with three segments.

#12. **Clypeus transection**

(0) from flat, in the same plane as the lower face, to weakly but evenly convex;

(1) basally slightly swollen then slightly concave apically, so in median longitudinal section it would be slightly sinuous (Fig. 44*);

(2) strongly convex basally and remainder protruding from the face, beak like (cf. *Helictes*-group in Orthocentrinae);

(3) convex and thus thickened all the way (cf. *Ophion*, *Xestopelta*);

(4) thickened in middle, thin at the apex, thus protruding first then concave (cf. *Collyria*);

(5) basal part flat in the same plane as the lower face, apical part protruding , so clypeus overall looks slightly concave (cf. *Heteropelma*);

(6) strongly and evenly protruding  from the base to the middle, then abruptly broken with apical part  almost completely horizontal, thus triangular in the profile (cf. *Xenoschesis*, Fig. 4^S^);

(7) flat in the same plane as the lower face but at the very apex thickened.

#13. **Clypeus shape viewed from front and division**

(0) entire, simply transverse to sublenticular, clearly wider than long;

(1) entire, small and subquadrate;

(2) transversely divided, with apical part smaller or subequal to the basal part, two parts more or less equally sclerotised (*Xanthopimpla*, *Lissopimpla*, *Hellwigia*);

(3) transversely divided, with apical part larger and less sclerotize than the basal part (some Labeninae and Xoridinae);

(4) transversely divided, two parts equally sclerotised with apical part transverse (in tiny Cretaceous ichneumonid, 3311/856b, Fig. 5^S^);

(5) entire, larger than in state 1, about as long as wide (cf. *Helictes*-group Orthocentrinae);

(6) narrow longitudinal, median part protruding and elongate, lateral parts in plane with face (cf. Hybrizontinae).

#14. **Apical margin of clypeus**

(0) simple, truncate to slightly convex or slightly concave, without tooth/teeth;

(1) slightly convex and medially pointed;

(2) with small acute lateromedian denticles;

(3) with central part weakly sclerotized and more or less notched, laterally bilobed (Figs. 23–25*);

(4) trilobed (Fig. 6^S^);

(5) strongly convex without a lobe (cf. *Scutellator*, *Procinetus*, Fig. 7^S^);

(6) with two lateral tooth-like protrusions (cf. *Heteropelma*, Fig. 8^S^);

(7) as state 0 but apical margin weakly sclerotized;

(8) convex, medially thickened (cf. *Astiphromma*);

(9) laterally bilobed, central part broadly truncated (Fig. 9^S^).

Note: State 3 includes also bilobed clypeus with apical tubercula in the middle (cf. *Coleocentrus*, Fig. 3^S^). It differs from the state (4),‘trilobed clypeus’, in tubercula being more or less dorsal to the apical margin of clypeus, which is visible and straight. In contrast, state 4 considers that the whole margin of the clypeus is protruding in the middle (also when double margin, cf. *Phorotrophus*, Fig. 6^S^). In case where it was difficult to delimit the two states (cf. *Arotes moiwanus*, Fig. 1^S^), character coded as polymorphic (3, 4).

#15. **Apical tubercula of clypeus**

(0) more or less round and small;

(1) enlarged and wide.

Note: Coded only when the character #14 coded as state (3) or (3, 4).

#16. **Apical margin of clypeus**

(0) single margin;

(1) double margin (cf. *Phorotrophus*, Fig. 6^S^).

Note: State (1) is synapomorphy for the tribe Acaenitini of the subfamily Acaenitinae.

#17. **Sexual dimorphism of clypeus**

(0) similar in both sexes;

(1) that of male centrally convex or biconvex, that of female much flatter;

(2) that of male centrally more concave, i.e. apical part more protruding.

#18. **Clypeal margin vestiture**

(0) with small scattered setae or lacking setae;

(1) with regularly spaced strong setae (cf. *Thymaris*).

Note: State (1) is often used as synapomorphy for the subfamily Tryphoninae, although it does occur in some *Lycorina*.

#19. **Labrum**

(0) mostly concealed below clypeus, at most narrowly visible, weakly sclerotized;

(1) clearly exposed below clypeus, but not strongly sclerotized (Fig. S^7^);

(2) exposed below clypeus, as strongly sclerotized (cf. Labeninae).

#20. **Tentorial pits**

(0) very small, indistinct;

(1) middle size, length between 1/3 and 1/2 of the breadth of the first flagellomere;

(2) large, longer then 1/2 of the breadth of the first flagellomere.

#31. **Face**

(0) separated from clypeus, flat to slightly convex, with a weak median swelling;

(1) separated from clypeus, biconcave lateromedially, with a central and pair of lateral ridges;

(2) separated from clypeus by groove, and strongly protruding (cf. *Strongylopsys*);

(3) not separated from clypeus, flat or with weak median swelling;

(4) not separated from clypeus, strongly inflated, very convex in profile (cf. *Acaerataspis*);

(5) not separated from clypeus, strongly protruding at the level of toruli, the lower part in profile more or less straight or at most slightly convex (cf. *Orthocentrus*-group);

(6) not separated from clypeus, whole surface protruding like a plate (cf. *Schyzopyga*);

(7) with very deep emarginations dorsally and laterally of clypeus (cf. *Dialipsis*);

(8) clypeus and lower face together forming a nose-like protrusion (cf. *Zagryphus*)

(9) shaped like a shield (cf. *Metopius*).

#32. **Upper face process**

(0) absent;

(1) upper face produced into a triangular process that extends between or over the bass of the antennae (cf. most Metopiinae).

#33. **Inner margin of eye**

(0) straight or weakly concave opposite antennal socket (Fig. 29*);

(1) with a deep, more or less V-shaped invagination opposite antennal socket (Fig. 30*).

#34. **Eye inner margin**

(0) more or less parallel;

(1) strongly converging ventrally, eyes enlarged.

#35. **Eye size relative to head height** [ordered]

"Continuous character transformed to 6 states (0–5). The character measured in lateral view as ratio of maximal eye to maximal head height; head height was measured from the base of mandible to the vertex (see Fig. 4^S^).

#36. **Colour of frontal orbit of female**

(0) concolourous with remainder of frons;

(1) with pallid markings or completely, contrasting strongly with remainder of frons (Fig. 7^S^).

#37. **Surface of eye**

(0) glabrous or with inconspicuous hairs;

(1) with long conspicuous pubescence (cf. *Collyria trichophtalama*).

#38. **Size of ocelli**

(0) normal size, separate from each other and eyes by more than their diameter, on one plane with vertex;

(1) enlarged, closely spaced, on raised triangular area.

Note: State (1) occurs in the night active ichneumonids e.g., *Ophion*, *Netelia*.

#39. **Facial pubescence of male**

(0) similar to that of female;

(1) very dense, obviously more pubescent than that of female.

#40. **Gena**

(0) smooth, punctured or shagreened;

(1) with numerous denticules.

Note: State (1) is synapomorphic for Poemeninae.

#41. **Occipital carina***

(0) more or less complete, dorsally evenly convex or slightly flattened, normally raised (Figs 38, 39, 40*);

(1) broadly absent dorsally, but ventrally present (Fig. 37*);

(2) entirely absent;

(3) more or less complete, dorsally evenly convex, flange-like;

(4) more or less complete, dorsally strongly dipped (Fig. 36*), weakly raised, sometimes very weak or obsolescent near midline, but then with upper end of lateral portion curved downwards.

#44. **Head in profile at vertex***

(0) moderately long, weakly and evenly rounded down to occipital carina;

(1) steeply declivous behind ocelli but surface flat or slightly convex;

(2) long and convex, usually with occipital carina rather low on head;

(3) precipitously declivous behind posterior ocelli, concave;

(4) raised or horizontal, then abruptly declivous behind occipital carina;

(5) like the state (1) but occipital carina very low on head;

(6) very short, distance between lateral ocelli and occipital carinae less than the diameter of the ocelli (cf. Anomaloninae).

#45. **Occipital notch***

(0) absent;

(1) present, ranging from a deep cleft with raised lobes laterally to a weak impression just above the foramen (Figs. 35, 38, 39, 41*).

#46. **Foramen magnum: flange***

(0) with a narrow flange dorsally;

(1) with a broad dorsal flange.

#47. **Foramen magnum: shape***

(0) subcircular;

(1) laterally expanded.

#48. **Foramen magnum: location***

(0) in a broad shallow concavity (Fig. 35*);

(1) in a deep almost circular pit, surrounded by a weak auxiliary carina (Fig. 36*).

#49. **Apical flagellomere modification**

(0) without modification;

(1) with some thickened setae;

(2) with cluster of modified setae;

(3) dense cluster of thickened and truncate structures forming a patch, no fusion of the structures, fused in the middle, or forming a flat surface;

(4) the last flagellomere with apex pointed;

(5) the last flagellomere itself with apex modified so that resembles a sting.”

#50. **Pedicel size***

(0) conspicuously smaller than scape, not inflated, and only of slightly greater diameter than 1st flagellomere (Fig. 33*);

(1) strongly inflated, almost as broad as scape and distinctly broader than 1st flagellomere (Fig. 34*).

#51. **Scape shape**

(0) conspicuously shortened so laterally not longer than pedicel and shorter than wide (cf. *Metopius*);

(1) 1­–1.5 times longer than wide, truncation oblique;

(2) elongate to cylindrical, >1.8x as long as wide with truncation at about 60–90° to longitudinal axis (cf. *Orthocentrus* genus-group).

#52. **Scape with extended membranous area**

(0) absent;

(1) dorsally present (cf. *Megastylus*);

(2) ventrally present, with dorsal side of scape inflexed under the ventral side (cf. *Xanthopimpla rhopalocera*).

#53. **Flagellum colour***

(0) without a median white band;

1. with a median white band.

#54. **Male flagellum central flagellomeres**

(0) lacking tyloids;

(1) with elliptical tyloids (cf. *Sussaba cognata*);

(2) with linear tyloids (cf. *Promethes sulcator*);

(3) with concave tyloids.

#55. **Ratio of antenna and fore wing length** [ordered]

Continuous character transformed to six states (0–5). Antenna length measured including scapus and pedicel. Fore wing length measured from the wing base to the most distal point.

#56. **Number of flagellomeres** [ordered]

Continuous character transformed to six states (0–5).

#57. **Antenna thickness**

(0) more or less of even thickness;

(1) evenly tapering towards the base, broadest flagellomeres close to the antennal tip;

(2) abruptly enlarged close to the apex, including only a handful of segments;

(3) evenly tapering towards the apex, last segments conspicuously narrower than the basal ones.

#58. **Modifications between antennal sockets and median ocellus**

(0) absent or with low, inconspicuous carinae;

(1) strong oblique pyramidal thickening ;

(2) longitudinal keel between impressions (Townesitinae);

(3) strongly raised keel between antennae that can be complete (cf. *Colpotrochia*, Fig. 10XA) or partial (cf. *Metopius*);

(4) two parallel low longitudinal ridges;

(5) rather low longitudinal ridge that extends more or less to a lobe at the level of antennal sockets and reaching at least ventral margin of antennal sockets (Fig. 10XB);

(6) raised keel between antenna that continues to a low ridge towards lower face (the dorsal part same shape as in state (3) when complete) (Fig. 10XC);

(7) strong impression between toruli and behind each torulus, the central impression separated from the side ones by oblique ridges (Fig. 10XD).

Note: It is possible that the state (2), which occurs in the fossil subfamily Townesitinae, is equivalent to state (3) or (5) or (6), but we could not asses this from the original descriptions and examinations of the fossils.

#59. **Pronotum in profile** [ordered]

(0) short and deep, <0.6x as long as deep, with collar (if present) very close to mesoscutelar margin (Fig. 48*);

(1) moderately long, 0.8–1.0 times as long as deep, collar not close to mesoscutum (Figs 47, 52*);

(2) long, >1.1 times as long as deep, collar not close to mesoscutum but mediodorsal region still rather short and furrowed (Fig. 50*);

(3) long, but with mediodorsal region horizontal and lengthened (Fig. 51*).

#60. **Anterior margin of pronotum***

(0) simple;

(1) mediodorsally reflexed, and directed backwards as a strong pointed tubercle (Figs 47, 48, 50*);

(2) with a very weak posteriorly directed median indentation."

#61. **Modification posterior to anterior margin of pronotum**

(0) vertical part not or only slightly thickened, horizontal part simple;

(1) raised as a distinct horizontal prominence, but this prominence still fused with remainder of pronotum;

(2) with horizontal ‘shelf ’not laterally closed or laterally closed to form a pocket-like structure (Fig. 51*);

(3) with one medial and two lateral ridges (cf. *Xorides praecatorius*);

(4) with one medial ridge (cf. *Poecilocryptus*);

(5) vertical part not or only slightly thickened, horizontal part thickened anterior and with clear transversal impression posterior;

(6) vertical part longitudinally impressed forming a channel (cf. *Rhyssa persuasoria*);

(7) vertical part thickened anteriorly and with a longitudinal channel, posterior with transverse impression (cf. *Diacritus*);

(8) with two lateral longitudinal ridges enclosing triangular area (cf. *Homolobus*, Braconidae)."

Note: State (3) in *Xorides praecatorius* is similar to the state (8) in braconid, potentially supporting basal position of xoridine in ichneumonids.

#62. **Epomia**

(0) complete, forming a weak ridge shaped like a figure 7 or reduced with only upper part present;

(1) entirely absent (Figs 49, 51*);

(2) with lower part long, extending almost till the ventral margin of the pronotum, while pronotum clearly raised below the epomia;

(3) with lower part long and strongly raised as a flange;

(4) with epomia very strong and expanded dorsally into a tooth;

(5) as state (2) but pronotum not raised below the epomia, instead anterior pronotal margin protruding into a tooth.

#63. **Modification anterior around base of notaulus**

(0) absent;

(1) with a thickening or crest at the base of notauli (cf. *Lissopimpla*, *Xanthopimpla*);

(2) with a paired crest at the  anterior dorso-lateral part of propodeum (cf. *Acrodactyla degener*);

(3) with a short carina directly in front of and parallel to notaulus (cf. some Orthocentrinae);

(4) with strong protrusion of mesoscutum between notauli into paired lobes;

(5) with pronotal margin laterally swollen."

#64. **Pronotum and tegula**

(0) with a tiny sclerite (prepectus) present between hind corner of pronotum, mesopleuron and tegula, thus causing the pronotum to bow out a little around this sclerite;

(1) with hind corner of pronotum overgrowing this sclerite, but sclerite still partially visible (cf. *Apophua*);

(2) with hind corner of pronotum completely overgrowing this sclerite and sclerite not visible (cf. Metopiinae).

#65. **Propleuron**

(0) without a lateroventral posteriorly-projecting lobe;

(1) with a lateroventral posteriorly-projecting lobe.

#66. **Sculpture of mesoscutum**

(0) finely punctate;

(1) smooth and impunctate;

(2) very coarsely punctate, with punctures separated inter se by about their own diameter;

(3) coarsely and closely punctate, the punctures more or less contiguous;

(4) evenly shagreened and matt;

(5) with sharp transverse rugae;

(6) with strong punctures and longitudinal striae formed of confluent punctures (cf. *Armadillocryptus*).

Note: Sculpture is often not uniform across the surface thus we coded dominant sculpture.

#67. **Hairs on mesoscutum**

(0) evenly pubescent;

(1) glabrous, at most with a few hairs around anterior or position of notauli, these hairs short;

(2) glabrous, but with a few very long hairs along front and position of notauli, these hairs as long as diameter of 1st flagellomere;

(3) middle evenly pubescent, laterally more glabrous.

#68. **Lateral flange posteriorly***

(0) complete to anterior end of scutellum;

(1) evanescent, not reaching the scutellum.

#69. **Lateral flange postero-centrally***

(0) narrow, not or only barely broader than it is anteriorly;

(1) strongly broadened, at least twice as wide as it is anteriorly.

#70. **Notauli** [ordered]

(0) absent;

(1) shallow or vestigial;

(2) deeply impressed at least anteriorly.

#71. **Notaulus length**

(0) present only on the frontal, vertical part of mesoscutum;

(1) extending to half length of mesoscutum, more or less parallel;

(2) extending to half length of mesoscutum, strongly converging;

(3) extending posteriorly past centre of mesoscutum but not joining other notaulus;

(4) extending posteriorly past centre and joining either the other notaulus or a sunken, medial area (cf. *Diacritus*);

#72. **Lateral longitudinal carinae of scutellum**

(0) absent or only discernible on extreme anterior end;

(1) more or less complete to centre or beyond, but not reaching posterior margin;

(2) complete to posterior margin;

(3) complete and expanded posteriorly into two acute teeth (cf. *Metopius*)."

#73. **Scutellum In profile**

(0) more or less flat;

(1) a bit convex;

(2) pyramidal, i.e. very strongly convex in profile, sometimes even drawn into a point between base and middle of scutellum (more triangular pyramid), posterior surface long and evenly declivous;

(3) strongly convex in profile, posterior surface convex, rather short (cf. *Phorotrophus*);

(4) pyramidal in profile, but with acute highest point almost at the end, after steeply declivous (*Astiphromma dorsale* group);

(5) as state 2, but with carinae along the posterior slope (and at lower front corners—triangular pyramid);

(6) strongly pyramidal in profile, but also with the whole shape (cf. *Opheltes*);

(7) slightly convex with posterior part raised (cf. *Tymmophorus*)."

#74. **Dorsellum**

(0) normally flat to evenly convex;

(1) conspicuously short, sharply rounded and strongly declivous posteriorly (cf. *Camptotypus*);

(2) triangular in profile and pointed (cf. some Orthocentrinae);

(3) long and flat, with two low longitudinal carinae and small swelling posteriorly.

#75. **Mesopleuron centrally**

(0) rather flat or weakly convex, at most with a weak diagonal impression delineating a convex area posterodorsally (Fig. 54*);

(1) with a strong horizontal impression reaching from mesopleural sulcus forward to near upper end of epicnemial carina;

(2) very strongly convex so that the body is much wider when viewed from above at height of mesopleuron than further back at the mesepimeron (cf. *Metopius*)."

#76. **Epicnemial carina**

(0) not curving anteriorly, vertical to around mid-height of pronotum (Figs 53, 54*);

(1) curving to anterior end of mesopleuron at around the mid-height of the pronotum;

(2) extending all the way to subtegular ridge;

(3) present ventrally only (not extending dorsally to ventral edge of pronotum);

(4) present only laterally;

(5) absent.

#77. **Mesopleural sulcus**

(0) angled opposite mesepisternal scrobe, usually with a shallow horizontal impression extending from this angulation to the scrobe (Fig. 53*);

(1) more or less straight with a shallow horizontal impression extending from this angulation to the scrobe;

(2) more or less straight with isolate puncture instead of the horizontal impression (Fig. 54*).

#78. **Mesosternum behind fore coxae**

(0) simply transverse;

(1) with anterior transverse carina produced forwards in a broad blunt angulation;

(2) with anterior transverse carina produced forwards in a narrow blunt angulation, with mesosternum protruding in its middle and forming a vertical ridge;

(3) produced forward into a large lobe;

(4) with a pointed tubercule in the middle.

#79. **Posterior transverse carina of mesosternum**

(0) absent;

(1) absent in front of the middle coxae, present otherwise;

(2) complete;

(3) short and present only in the middle, in-between the two mid coxae;

(4) clearly present laterally, but absent in front of the mid coxa, in between coxae absent (or fused?) with anterior transverse carina;

(5) absent laterally but present as extended flanges in front of the mid coxae and in-between."

#80. **Sternaulus**

(0) absent or present less than 0.7x length of mesopleuron;

(1) present, greater than or equal to 0.7x length of mesopleuron.

#81. **Metanotum**

(0) short, so that the constriction between metanotum and propodeum is very close after the dorsellum (postscutellum);

(1) elongate, so that the constriction is clearly removed from the dorsellum, by about 0.2­­–0.3x the length of the propodeum.

#82. **Metapleuron** [ordered]

(0) clearly higher than long (cf. Diplazontinae);

(1) about as long as wide;

(2) almost 2x as long as wide.

#83. **Submetapleural carina**

(0) complete, anterior section unmodified to slightly broadened (cf. *Pimpla*);

(1) complete, anterior section abruptly broadened into a lobe (cf. *Lissopimpla*);

(2) anteriorly present, not-lobe like, posteriorly absent (cf. *Dolichomitus*);

(3) completely absent;

(4) anterior section more or less broadened, but carina not complete to posterior edge;

(5) complete, broadened on the whole length.

#84. **Propodeum in profile**

(0) from rounded to dorsally flattened, and with a posterior surface (Fig. 59*);

(1) evenly declivous (Fig. 60*);

(2) with postero-medial part elongated into a tube almost as long as propodeum itself.

#85. **Propodeum length** [ordered]

(0) clearly shorter than high (cf. Diplanzontinae);

(1) about as long as high (Figs 61, 62*);

(2) elongate, distinctly longer than wide, cylindrical.

#86. **Pleural carina** [ordered]

(0) mostly absent (only with indications at beginning and/or end);

(1) partially reduced or weak;

(2) present as distinct carina on whole length.

Note: The ichneumonid propodeum shows a lot of variation both between and within subfamilies. The ground plan or full set of carinae includes three longitudinal (median, lateral, and pleural) and two transverse carinae (basal and apical) (see Fig. 11X). In addition, the juxacoxal carina of the metapleuron is also developed partly or completely in a variety of taxa. There is a variety of patterns in which the ground plan carination of the propodeum is modified. In many groups, the carination is completely or partly reduced, and this character seems to change rather quickly, i.e., even within subfamilies and certain genera. The overall completeness of the carination seems only partly related to the size of the specimens, but certainly has a phylogenetic component. Some subfamilies only possess specific carinae, others follow the complete carination e.g., if the propodeum is shortened, as in Diplazontinae (see Fig. 12X), the petiolar area is often fused with the areola.

#87. **Juxacoxal carina** [ordered]

(0) mostly absent (only with indications at beginning and/or end);

(1) partially reduced or weak;

(2) present as distinct carina on whole length.

Note: Carina low on metapleuron and usually absent. See Fig. 11^S^ and note under the character #86.

#88. **Longitudinal carinae**

(0) absent;

(1) only median longitudinal carinae present;

(2) only lateral longitudinal carinae present;

(3) both medial and lateral longitudinal carinae present.

Note: See note under the character #86.

#89. **Anterior transverse carina**

(0) complete (medially and sublaterally);

(1) medial abscissa present, sublateral abscissae (= costulae) absent;

(2) median abscissae absent, sublateral abscissae present;

(3) completely absent.

Note: Condition of the lateral abscissa was not coded because the presence of the spiracle makes scoring the condition in this region problematical. See Fig. 11^S^ and note under the character #86.

#90. **Anterior transverse carina shape**

(0) angled;

(1) forming more or less a smooth arc.

Note: if carina absent or incomplete so that angulation/curvature could not be scored, characters is coded as NA. See also Fig. 11^S^ and note under the character #86.

#91. **Posterior transverse carina of propodeum**

(0) complete (medially and sublaterally);

(1) medial abscissa present, sublateral abscissae absent;

(2) median abscissa absent, sublateral abscissae present (areolar area confluent with petiolar area);

(3) completely absent/ indistinguishable. "

#92. **Posterior transverse carina shape**

(0) angled;

(1) more or less a continuous arc.

Note: If carina absent or angulation uncertain, coded as NA. See also Fig. 11^S^ and note under the character #86.

#93. **Spiracle** [ordered]

(0) subcircular to oval, < 2.0 times as long as wide (Fig. 59*);

(1) elongately oval >2.0 but less than 3.5 times as long as wide (Fig. 60*);

(2) elongately elliptical >3.5 times as long as wide (Fig. 63*).

#94. **Spiracle position**

(0) separated from pleural carina by about minimum diameter of spiracle or more (Fig. 63*);

(1) separated from pleural carina by less than minimum diameter (Fig. 62*).

#95. **Posterolateral angle of propodeum**

(0) evenly rounded;

(1) swollen (cf. *Zaglyptus*, Fig. 59*).

#96. **Modifications on dorsal postero-lateral surface of propodeum**

(0) without apophyses (Fig. 56*);

(1) with subconical apophyses present at position of junction of posterior transverse carina and lateral longitudinal carina (Figs 57, 63*);

(2) with posterior transverse carina elevated into a flange on points where lateral longitudinal carina would have joined;

(3) with flanges present at the position of junction of posterior transverse carina and lateral longitudinal carina (cf. *Opheltes*)."

#97. **Modifications on dorsal postero-medial surface of propodeum**

(0) without modifications;

(1) with a strong single median tubercle (Figs 57, 63*);

(2) with posterior transverse carina elevated into a flange that forms a shelf;

(3) with hind margin of propodeum extended towards metasoma.

#98. **Hind margin of propodeum**

(0) simple;

(1) extended and covering anterior part of metasomal first tergit.

#99. **Propodeal surface sculpture**

(0) smooth and shiny, without reticulation between carinae and at most with some weak punctures where hairs are inserted  (Fig. 55*);

(1) mostly covered by reticulation that obscures carinae;

(2) with distinct punctures;

(3) rugulopunctate;

(4) with weak punctures, distance more than their diameter;

(5) evenly shagreened and matt without distinct punctures;

(6) with strong wavy pattern between carinae (cf. *Armadillocryptus*)."

#100. **Metasternum centrally**

(0) medially flat with a weak median longitudinal groove;

(1) with a raised central tubercle;

(2) with a raised anterior longitudinal crest;

(3) with a median groove that becomes a circular hole in the front.

#101. **Metasomal foramen ventrally**

(0) ventrally broadly U-shaped to a somewhat rounded V-shape;

(1) deeply V-shaped.

#102. **Metacoxal cavity**

(0) dorsal margin is dorsal to the ventral margin of metasomal foramen magnum;

(1) dorsal margin just on height of lower margin of metasomal foramen magnum;

(2) dorsal margin is clearly ventral to the ventral margin of metasomal foramen magnum.

#103. **Intercoxal carinae**

(0) weak, usually with vestiges meeting ventral rim of metasomal foramen;

(1) with posterior end of carina meeting its fellow medially and forming a short but strongly raised medial carina that reaches back to the rim of the foramen (Fig. 87*);

(2) abruptly angled posteriorly to meet its fellow and not joined to rim of foramen, instead making a sharp carina between or in front of coxal foramena.

Note: To code this character one often has to detach hind legs from propodeum. We thus did not code this character when the specimens had to be left intact i.e., in case of museum specimens on loan. This character was never visible in fossil specimens.

#104. **Apodeme of hind coxa**

(0) with lateral apodeme not or barely twisted;

(1) with lateral apodeme twisted about 60–75°, and with a corresponding deep notch in lateral margin of coxal socket (cf. Fig.63* which has a notch with Fig. 61* where one is not present).

#105 **Propodeal denticles***

(0) absent;

(1) present (Fig. 64*).

#106. **Fore** **femur of male**

(0) ventrally unspecialized;

(1) anteriorly concave beneath, and with a central blunt prominence.

#107. **Fore femur of female**

(0) ventrally unspecialized, more or less convex or slightly flattened;

(1) with a longitudinal concavity (which may sometimes be biconcave).

#108. **Fore tibia of female**

(0) simple;

(1) enlarged and inflated;

(2) slender and bowed to fit around angularly produced femur.

#109. **Fore tibia**

(0) without spines;

(1) with scattered long and stronger spines;

(2) bearing stout short conical spines set in pits.

#110. **Apex of fore tibia**

(0) without tooth;

(1) with a distinct tooth on dorsal margin (cf. Ctenopelmatinae).

#111. **Fore leg of female with 5th tarsomere**

(0) slender, of similar thickness to other tarsomeres;

(1) exceptionally swollen."

#112 **Legs elongate**

(0) front femur including trochanter <0.3x forewing;

(1) >0.3x forewing.

#113 **Tarsal claws of female**

(0) all simple;

(1) all modified;

(2) hind simple, others modified;

(3) fore modified others simple.

#114 **Tarsal claws of female: modification type**

(0) basal lobe;

(1) tooth;

(2) comb.

Note: Codded as NA if all claws simple.

#115. **Hind claws: apex**

(0) simply pointed;

(1) bevelled.

#116. **Mid coxa of female: inner surface**

"(0) simply convex;

(1) with shallow groove;

(2) with shallow groove bordered by sharp parallel carinae."

#117. **Mid coxa of female: outer surface**

(0) simply convex, unspecialized;

(1) with convex impressions so it appears to have bumps.

#118. **Spurs on mid tibia**

(0) two spurs;

(1) one spur.

#119. **Apex of middle and hind tibiae**

(0) with common area of insertion for spurs and basitarsus;

(1) with sclerotized bridge separating insertion areas (cf. Cremastinae).

#120. **Hind coxa proportions** [ordered]

(0) short and strongly inflated, deeper than long;

(1) of normal dimensions, slightly longer than deep;

(2) elongate and slender, 1.5–3 times as long as deep;

(3) extremely elongate, > 3.5x as long as deep.

#121. **Hind coxa posteriorly**

(0) evenly rounded;

(1) bluntly angled on ventrolateral edge.

#122. **Hind coxa of female: inner surface**

(0) simply convex;

(1) with shallow groove;

(2) with shallow groove bordered by sharp parallel carinae;

(3) with furrow covered with grossulariform setae.

#123. **Hind femur ventrally**

(0) simple;

(1) with a blunt tooth.

#124. **Hind leg femur dimension** [ordered]

(0) conspicuously thickened, up to 2.5x as long as deep;

(1) normal dimensions, 3–5x as long as deep;

(2) elongate, > 5x as long as deep.

#125. **Hind tibial spurs**

(0) long and slender, of normal dimensions, pointed apically;

(1) short and stout, the inner spur with a broad, truncated edge;

(2) short and stout, but with tips normal, even though often quite curved;

(3) inner long and slender, outer short and very stout.

#126. **Hind tibia pubescence** [ordered]

(0) covered with a uniform thickness of setae;

(1) with uniform setae and sparse, stouter spines (spines much slender compared to the state 2 spines, not very obvious among setae);

(2) with uniform setae and sparse, very stout conus shaped spines (spines very conspicuous, setae very inconspicuous) (cf. *Xanthopimpla*).

#127. **Hind tibia colouration**

(0) uniformly red, brown, orange or yellow;

(1) orange or brown with apex darkened;

(2) white with apex darkened;

(3) black-white-black banded;

(4) orange or yellow with base and apex darkened;

(5) red, brown, orange with base lighter;

(6) black with a narrow light base;

(7) orange or yellow with base darkened;

(8) longitudinally split: orange in posterior, brown in anterior half.

#128. **Hind tibia shape**

(0) normal, parallel sided to evenly tapered or a bit enlarged, so that base is distinctly thinner;

(1) very strongly enlarged, becoming tubular.

#129. **Fringe of parallel setae on *inner* side of apex of hind tibia**

(0) absent, only with a few hairs apically which are very similar as the hairs on the surface;

(1) present as a fringe of parallel setae which are very similar to the main pubescence on the hind tibia;

(2) present as a very dense fringe of parallel bristle which clearly differ from the main pubescence of the tibia and from the fringe on the outer surface.

#130. **1st tarsomere dimension**

Continuous character transformed to six states (0–5). Measured as ratio of 1^st^ trarsomere length to width.

#131. **Hind leg with 2nd to 4th tarsomere of female ventrally**

"(0) with pubescence that is similar to the dorsal pubescence;

1. with strong stout spines that are obviously thicker than the dorsal pubescence (Fig. 92*)."

#132. **Hind leg with 4th tarsomere**

(0) apically more or less evenly truncated;

(1) apically obliquely truncated, so that inner side overhangs base of the 5^th^ tarsomere.

#133. **Hind tarsus of female with orbicula**

(0) short and moderately broad, 2.5–3.5x as long as wide;

(1) very slender and bar-like, > 3.5x as long as wide (Fig. 91*);

(2) very short, only about 2x as long as wide.;

(3) broad and expanded anteriorly, almost as long as wide (cf. *Heteropelma*).

#134. **Hind tarsus with pulvillus**

(0) of normal dimensions, not projecting beyond apex of claw;

(1) enlarged, projecting conspicuously beyond apex of claw (Fig. 93*).

#135. **Poison vesicle on hind tarsal claw**

(0) without any trace of a membranous poison vesicle;

(1) with a small membranous vesicle on inside of apical tooth of male;

(2) with a small membranous vesicle in male and female;

(3) with a large membranous vesicle in both sexes.

#136. **Spatulate bristle on claws**

(0) without a spatulate bristle;

(1) with a long conspicuous spatulate bristle, the apex of which (at rest) is pressed against the pointed apex of the claw.

#137. **Vein 3rs-m**

(0) absent;

(1) spectral ~ 100%;

(2) with one bulla;

(3) with two bullae;

(4) without bulla.

#138. **Areolet shape**

(0) quadrate-oblique to triangular, with 2+3M longer than 4M, 2Rs more than 0.3x 3M (Fig. 13A^S^);

(1) rhombic, all sides almost equal (Fig. 13B^S^);

(2) pentagonal, with +/- even sides (Fig. 13C^S^);

(3) as state (0), but 2Rs less than 0.3x 3M (Fig. 13D^S^);

(4) widened pentagonal, > 1.5x as wide as deep (Labenopimplinae type, Fig. 13E^S^);

(5) Braconidae condition, not proper areolet, but cell that is of similar size to other cells (widened quadrate, 2+3M and 4M together, 2m-cu absent, 3Rs long) (Fig. 13F^S^);

(6) strongly petiolate areolet, petiolous longer than the width of surrounding veins (Fig. 13G^S^);

(7) quadrate or pentagonal with 2Rs more than 2x 2+3M (Fig. 13H^S^);

(8) with 2Rs absent, thus areolet open towards the inside (cf. Ophioninae, Fig. 13I^S^);

(9) quadrate-asymmetric, but not oblique: with 3M shorter than 4M, and 2Rs of similar length as 3M (usually rather big areolet, cf. many Metopiinae, Fig. 13J^S^).

Note: Condition of open areolet (3rs-m absent) is covered by state (0) in the character #137. The shape of such areolet is often still discernible base on the length of the remaining veins (4M usually darker than 5M and the whole vein section (4M+5M) often broken where 4M is ending), but if this is not the case shape coded as polymorphic. Areolet which is petiolate with petiolus shorter than the width of the surrounding veins was coded as state (0), and one with petiolus as long as the width of surrounding veins was codded as polymorphic (0,6).

#139. **Vein 2m-cu**

(0) present (Fig. 1);

(1) absent (cf. *Homolobus*, Braconidae, Fig. 13F^S^).

#140. **Vein 2m-cu: bullae number**

(0) with two bullae separated with only trace of the vein or with longer part of the vein;

(1) with a single bulla;

(2) entirely spectral.

#141. **Vein 2m-cu: size of bullae**

Continuous character transformed to six states (0–5). Size of bulla was measured as percentage of 2m-cu length that bulla occupies. When two bullae present, their percentages were summed.

#142. **Vein 2m-cu: shape**

(0) straight;

(1) more or less evenly bowed outwards;

(2) with an outwards facing zig-zag to sinusoidal;

(3) bowed or angled inwards.

#143. **Antero-medial forewing flexion line basally**

(0) splitting anterior to M and anterior fold running anterior to M (forming bulla indicated by arrow), posterior fold posterior to M (cf. Braconidae)

(1) anterior fold, if present, running posterior to M (creating bulla(e) in vein 2m-cu indicated by arrow(s)) (cf. Ichneumonidae).

Note: In braconids (state (0)), the basal vein of the areolet has a bulla which never happens in Ichneumonidae (state (1)).

#145. **Fore wing cell 1M+1R1: pubescence**

(0) uniformly hirsute;

(1) with small to large glabrous area that may have free sclerites (cf. *Leptophion*).

#146. **Fore wing vein 4Rs**

(0) almost straight;

(1) proximally arched;

(2) distally arched;

(3) sinusoidal;

(4) evenly arched towards the inside of 2R1 cell.

#147. **Fore wing cell 1M+1R1: division**

(0) completely divided (vein 1Rs+M complete to vein 1Rs&1M);

(1) not completely or not at all divided.

Note: In braconids the 1M+1R1 cell is always *completely* divide (state (0)) while that is never the case in Ichneumonids.

#148. Remaining of the vein 1Rs+M (ramullus) [ordered]

(0) absent;

(1) present, at most as long as the width of the surrounding veins;

(2) present, longer than the width of the surrounding veins and at most reaching till half of the way to M.

Note: coded as NA when character #147 coded as state (0).

#149. **Fore wing vein 1cu-a (nervullus position)** [ordered]

(0) emerging anterior of the level of 1M ("antefurkal");

(1) at level of 1M ("interstitial");

(2) emerging posterior of the 1M ("postfurkal");

(3) extremely posterior so that vein 1Cu is > 0.8x length of vein 2Cu (some Brachycyrtinae).

Note: The limit for the state definitions was width of the surrounding veins i.e., if vein 1cu-a was not exactly at the level of 1M but still less than one width of the surrounding veins anterior or posterior from it, the state was coded as state (1). When this distance was around the width of the surrounding vein state was coded as polymorphic, (0,1) or (1,2).

#150. **Pterostigma dimensions** [ordered]

Continuous character transformed to six states (0–5). Measured as ration of maximal length to maximal depth.

#151. **Ratio of Pterostigma to 1R1 length** [ordered]

Continuous character transformed to six states (0–5).

#152. **Fore wing cell 2R1 dimensions** [ordered]

Continuous character transformed to six states (0–5). Measured as ration of maximal length to maximal depth.

#153. **Fore wing 5M vein** [ordered]

(0) tubular through the whole length, same colour as or slightly lighter than the colour of the surrounding veins;

(1) vein partly spectral;

(2) vein through the whole length spectral;

(3) absent.

Note: when assessing this character the most distal 10% of the vein was not taken into account since this portion of the vein is almost always spectral or absent. In addition, the most proximal part of the vein, at most as long as the width of surrounding veins, was allowed to be tubular in state (2) and tubular or spectral in state (3).

#154. **Ratio of 2Cu to 1M+1Rs length** [ordered]

Continuous character transformed to six states (0–5).

#155. **Ratio of 2Cu to r-rs length** [ordered]

Continuous character transformed to six states (0–5).

#156. **Fore wing 1m-cu&2Rs+M vein**

(0) straight;

(1) arched;

(2) angled;

(3) with a rounded hump;

(4) distal bow very strong.

#157. **Fore wing vein 4Cu vein: position relative to junction of 2Cu and 3Cu** (positon of postnervullus) [ordered]

(0) 0–0.25;

(1) 0.25–0.5;

(2) 0.5–0.75;

(3) 0.75–1;

#158. **Fore wing length**

Continuous character transformed to six states (0–5). Measure from the base of the fore wing to the most distal point.

#159. **Costa+Subcosta & Radius**

(0) fused or at least very close to each other, no membrane between visible;

(1) some distance apart, with membranous area between.

Note: All crown group ichneumonds have state (0).

#160. **Hind wing vein M+Cu: shape**

(0) straight;

(1) curved distally;

(2) curved middle;

(3) slightly curved through whole length.

#161. **Hind wing vein M+Cu: conditon**

(0) completely tubular;

(1) basal 0.6 spectral.

#162. **Hind wing vein 2Cu position relative to junction of M+Cu and 1Cu** (position of nervellus) [ordered]

(0) 0–0.25;

(1) 0.25–0.5;

(2) 0.5–0.75 ;

(3) 0.75–1.

#163. **Hind wing vein 2Rs** [ordered]

(0) vein tubular through the whole length, same colour as or slightly lighter than the colour of the surrounding veins;

(1) vein partly spectral;

(2) vein through the whole length spectral;

(3) absent.

Note: see note for the character #153.

#164. **Hind wing vein 2Cu** [ordered]

(0) vein tubular through the whole length, same colour as or slightly lighter than the colour of the surrounding veins;

(1) vein partly spectral;

(2) vein through the whole length spectral;

(3) absent.

Note: see note for the character #153.

#165. **Hind wing ratio of 1Rs to rs-m length**

"Continuous character transformed to six states (0–5).

#166. **Hind wing vein 1rs-m**

(0) basal to separation of veins R1 and Rs (cf. Braconidae, Fig. 18 in Goulet & Huber 1993);

(1) opposite or apical to separation of veins R1 and Rs (cf. Ichenumonidae).

#167. **Hind wing *basal* hamuli: postion**

(0) distant from wing base on membrane or spectral vein;

(1) close to wing base on spur of tubular vein;

(2) absent, though in their place often slightly thickened hairs;

(3) large number of basal hamuli along entire margin, half grown over by more sclerotized and narrow wing membrane in front of Sc+R (cf. Hybrizontinae).

#168. **Hind wing *basal* hamuli: number** [ordered]

(0) one;

(1) two;

(2) three;

(3) four;

(4) eight.

#169. **Ground plan colour**

(0) black or brown;

(1) orange;

(2) yellow.

#170. **Face ground colour**

(0) same as ground plan colour in both sexes;

(1) same as ground plan colour in female, male with different colour;

(2) same as ground plan colour in male, female with different colour;

(4) different than ground plan colour in both sexes.

#171. **Metasoma** [ordered]

(0) depressed to cylindrical all the way;

(1) compressed from segment 5;

(2) compressed from segment 4;

(3) compressed from segment 3.

#172. **T1 lateral longitudinal carina (or crease)**

(0) more or less complete, above spiracle;

(1) more or less complete, below or at spiracle;

(2) more or less absent, at most a vestige anteriorly and/or posteriorly;

(3) more or less complete but far below spiracle, posteriorly abruptly curved upwards.

#173. **T1 glymmae** [ordered]

(0) absent;

(1) present, shallow not meeting at midline (although each glymma may join lateral sides of anterodorsal sulcus of T1) (cf. *Phytodietus*);

(2) deep, almost meeting at midline (cf. *Netelia*)."

#174. **S1 fusion apically**

(0) not fused to T1 (cf. *Rhyssa*);

(1) fused, but suture visible (cf. *Megarhyssa*);

(2) fused, suture not visible.

#175. **T1 dimensions** [ordered]

(0) subquadrate;

(1) elongate, from about 1.5–3.5x as long as posteriorly wide;

(2) very long and slender, > 4.0x as long as posteriorly broad.

#176. T1 Shape from above

(0) petiolate, clear separation of postpetiolus;

(1) parallel sided;

(2) evenly tapering to front.

#177. **T1 shape in profile**

(0) rounded basally or with a hump around mid length, thus in profile more narrow in the front than in the apical half;

(1) flat in profile, at most with a very weak rounding basally, thus of even thickness in profile;

(2) basally straight and sometimes continuously expanding in profile, and with an angle in second half (often when petiolate).

#178. **T1 constriction**

(0) no diagonal constriction around mid length of tergite;

(1) with a constriction showing as a diagonal groove on the sides becoming a transverse impression dorsally (cf. *Xorides*).

#179. **T1 and T2**

(0) separated by a normal joint, thus as flexible as T2 to T3;

(1) partially fused so that T2 cannot move independently (cf. *Metopius*).

#180. **T1 dorsal carinae: length** [ordered]

(0) absent;

(1) less than half length of tergite;

(2) more than half length of tergite.

#181. **T1 dorsal carinae: position**

(1) converging, but then still about as far apart as distance to lateral margin;

(2) very strongly converging, ending up very close together when they become parallel;

(3) on elongate T1, just parallel.

#182. **T1 spiracle**

(0) at or anterior to 0.6x length of segment;

(1) posterior to 0.6x length of segment.

#183. **T1 sculpture, dorsal surface in the posterior half**

(0) smooth and impunctate;

(1) finely punctate, punctures small and distant;

(2) very coarsely punctate, with punctures separated inter se by about their own diameter;

(3) coarsely and closely punctate, the punctures more or less contiguous (but no rugae);

(4) shagreened;

(5) with sharp transverse rugae more or less punctured in-between;

(6) with longitudinal parallel dense rugae and indistinct punctures (cf. *Ichneumon*);

(7) rugulopuntured, rugae more or less longitudinal, interrupted, with punctures in-between;

(8) shagreened with longutudinal rugae.

#184. **Laterotergite one in female**

(0) more or less absent;

(1) broad on almost entire length of tergite and membranous;

(2) short and triangular but with at least some colour (cf. *Dialipsis*);

(3) elongate triangular with some colour;

(4) triangular on almost entire length and membranous.

#185. **S1 length in female** [ordered]

(0) very short, <0.3;

(1) about 0.4–0.5 of length of tergite;

(2) very long, 0.6–0.9;

(3) almost till the end of tergit.

#186. **S1 shape in female**

(0) with a weak to strong median keel anteriorly;

(1) flat, without a median longitudinal keel;

(2) with a median anterior invagination;

(3) as state (0) but with additionally transversal carinae behind.

#187. **S1 central ornamentation in female**

(0) not ornamented;

(1) with a low rounded swelling;

(2) with a strong sharp transverse crest;

(3) with a pointed tubercle;

(4) with paired tubercles;

(5) with large swelling;

(6) with a strong protruding longitudinal swelling (cf. *Hyperacmus*);

(7) with a low round swelling and longitudinal crest (cf. *Allomacrus*);

(8) with paired round swellings;

(9) longitudinal swelling on almost whole of the S1 length.

#188. **S1 hind margin shape**

(0) from transverse to shallowly V-shaped;

(1) sharply V-shaped with a median notch;

(2) with a very deep cleft, almost bifid;

(3) with a median posteriorly directed point.

#189. **T2 length in dorsal view** [ordered]

(0) transverse to subquadrate;

(1) 1.3–2.5x as long as posteriorly broad;

(2) > 2.5x as long as posteriorly broad.

#190. **T2 medial longitudinal carinae**

(0) absent;

(1) present as two parallel carinae;

(2) present, fused in a single medial carina.

Note: Carinae on T2 occur only in some Metopinae and in the diplazontine genus *Enizemum*.

#191. **Thyridium**

(0) absent;

(1) present.

#192. **Thyridium location**

(0) simple, thyridium not sunken in deep transverse depression (‘gastrocoelus’);

(1) thyridium sunken (gastrocoelus) (Figs 67, 68, 69*);

(2) thyridium in impression surrounded by carinulae.

#193. **Thyridium shape and distance from the anterior margin of T2**

(0) present, ovoid and  less than one length or diameter distant from anterior edge of T1;

(1)  present ovoid  and greater than or equal to one length or diameter distant from anterior edge of T1;

(2) present transverse and  less than one length or diameter distant from anterior edge of T1;

(3) present transverse  and greater than or equal to one length or diameter distant from anterior edge of T1.

#194. T2 impressions

(0) simple, with no impression behind thyridium (Fig. 67*);

(1) with a shallow very oblique impression behind thyridium, this impression subtending an angle >45° to longitudinal axis of tergite (Fig. 66*);

(2) with a long deep oblique impression reaching back to second thyridium, this impression subtending an angle <40° to longitudinal axis of tergite (Fig. 65*);

(3) with a weak to moderately strong transverse impression, sometimes with this only present laterally and not meeting on mid-line;

(4) with both longitudinal and transverse impressions.

#195. **T2 sculpture centrally**

(0) smooth and polished, sometimes with a few isolated punctures (Fig. 68*);

(1) evenly shagreened and matt;

(2) with deep punctures (Fig. 69*);

(3) transversely aciculate;

(4) rugopunctate;

(5) finely microreticulate;

(6) with longitudinal striae at least in basal part;

(7) finely evenly punctured.

#196. **T2** **Anterior margin medially in cross-section**

(0) broadly rounded;

(1) with a sharp median longitudinal ridge;

(2) with a shallow median longitudinal concavity (Fig. 68*);

(3) with two parallel carinae which continue the mediodorsal carinae of T1."

#197. **T3–T4 ssculpture of posterior 0.2**

(0) not sculpturally differentiated from anterior part of tergite, at most with extreme posterior end of tergite impunctate;

(1) with sculpture differing from rest of tergite (Figs 65, 66, 69*), usually smooth and impunctate.

#198. **T3–T4 colouration ♀**

(0) uniformly coloured;

(1) with colour different only in the last 0.2 of the tergites;

(2) with colour different on more than 0.2 of the tergite;

(3) with pattern in the form of paired spots or connected spots.

#199. **Laterotergite two: dimensions** **♀** [female]

"(0) more or less absent;

(1) moderately broad, 0.25–0.4 times as wide as long, and strongly sclerotized;

(2) broad, >0.5 times as wide as long, and membranous."

#200. **Laterotergite two: crease** **♀**

(0) creased and curved under metasoma (cf. *Netelia*);

(1) not separated by a crease.

#201. **T2 spiracle position**

(0) on dorsal part or directly on crease;

(1) clearly below the crease.

#202. **T2 and T3 fusion ♀**

(0) separate, with flexion line allowing movement;

(1) fused, without a flexion line.

#203. **T3 centrally** **♀**

(0) more or less evenly convex, without strongly raised areas;

(1) with conspicuous lateromedian rounded swellings (Fig. 71*);

(2) with a median rhombic raised area (Fig. 72*);

(3) with a transverse, lozenge-shaped area, bordered by deep grooves, at least posteriorly;

(4) with diagonal grooves starting basally close to the middle and pointing towards posterior corners of tergite, although not always reaching them (cf. Glyptini in Banchinae);

(5) with lateromedian round swellings in basal half and subapical transverse impression, between which there is a basally pointing flat triangle outlined by strong grooves (cf. *Lycorina*).

#204. Laterotergite three: dimensions **♀** [ordered]

(0) more or less absent;

(1) moderately broad, 0.25–0.4x as wide as long, and strongly sclerotized;

(2) broad, > 0.5x as wide as long, and membranous.

#205. Laterotergite three: crease **♀**

(0) separated by a crease at least until the spiracle, posterior part can be without the creast and often turned under;

(1) not separated by a crease.

#206. **T3–T4 posterolateral corners**

(0) more or less rounded or right-angled;

(1) incised, so corner is acute;

(2) obliquely truncate;

(3) tergites extended backwards laterally, so that they are shorter medially (cf. *Phthorima*, *Campocraspedon* in Diplazontinae).

#207. **Laterotergite four: dimensions ♀** [ordered]

(0) more or less absent;

(1) moderately broad, 0.25–0.4x as wide as long, and strongly sclerotized;

(2) broad, > 0.5x as wide as long, and membranous.

#208. **Laterotergite foure: crease ♀**

(0) separated by a crease (at least basally), often turned under;

(1) not separated by a crease.

#209. **T7 size ♀** [ordered]

(0) conspicuously shorter (at most 0.6x as long as tergite VI);

(1) similar in size to tergite VI, sometimes partially retraced under it, but not very noticeably smaller;

(2) conspicuously longer than tergite VI.

#210. **T7 size** ♂ [ordered]

"(0) conspicuously shorter (ab most 0.6x as long as tergite VI), or retracted invisible;

(1) of similar size to or slightly smaller than tergite 6;

(2) longer and usually more strongly sclerotized than tergite 6."

#211. S7 sclerotization ♂

(0) flat and evenly sclerotized;

(1) weakly sclerotized with a median longitudinal ridge.

#212. Anterior sternites ♀

(0) without ovipositor guides;

(1) with tubercular ovipositor guides (cf. *Megarhyssa*).

#213. T8 Lower anterolateral corner ♀

(0) with a small pointed or finger like apodeme (Figs 95, 96, 97*);

(1) with apodeme enlarged (Fig. 94*).

#214. T8 shape ♀

(0) short;

(1) elongate without horn or boss (cf. *Odontocolon*);

(2) elongate with horn or boss (cf. *Megarhyssa greenei*);

(3) laterally elongate without horn or boss, dorsally with a hole through which T9 visible (cf. *Xenoschesis* in Ctenopelmatinae).

#215. **T9 division** ♂

(0) entire;

(1) medially longitudinally divided.

#216. **Separation between T8 and T9 ♂**

(0) laterally separated, with tergite VIII forming a lateral plate on the side of tergite IX (Fig. 99*);

(1) with tergites VIII and IX completely fused laterally, seemingly just a single tergite (Figs 100, 1019).

#217. **S8 shape ♂**

(0) more or less transverse and moderately sclerotized (Fig. 78*);

(1) strongly sclerotized, short, deeply convex, closing apex of metasoma at rest (Figs 79, 80*);

(2) weakly to moderately sclerotized and rather flat, but elongate, more or less closing apex of metasoma at rest (Figs 81, 82*);

(3) similar to state 0, but with a median emargination (very small one, so maybe it should go to 0);

(4) narrow, paddle-shaped (cf. some Diplazontinae)."

#218. **Apex of aedeagus ♂**

(0) subcylindrical;

(1) dorsoventrally depressed.

#219. **Gonoforceps ♂**

(0) simple;

(1) apically long and rod-like (cf. *Mesochorus*);

(2) very short, and inconspicous.

#220. **T9 shape ♀**

(0) from triangular to strap-like, immutably fused with preceding tergite (though the join is generally discernible because of differing sculpture patterns) and occupying an indentation in it;

(1) almost D-shaped, and positioned behind (rather than within) tergite 8, the join between the two very weakly sclerotized;

(2) separated (some outgroup);

(3) forming a cornus (some outgroups);

(4) within the cavity of T8 (cf. *Xenoschesis* in Ctenopelmatinae).

#221. **Subgenital plate (hypopygium) shape ♀**

(0) transverse (wider than long when viewed from below), with posterior margin simple and more or less straight (not convex), in profile inconspicuous or barely visible (cf. Pimplini);

(1) transverse, with posterior margin simple and more or less straight (not convex), in profile square or rectangle, more conspicuous (cf. Campopleginae and probably most of the other groups with strongly compressed metasoma);

(2) elongate (longer than wide), posterior margin simple and convex, in profile small, more or less triangular but with the longest side rounded (cf. *Clistopyga*);

(3) viewed from below as wide as long or slightly elongate, posterior margin simple and slightly convex, in profile forming equilateral triangle or if scalene triangle, then the longest side straight (cf. Mesochorinae);

(4) sub-quadrate to elongate, posterior margin convex with median notch (cut), in profile diverse with lower margin simply straight or broken at the place where the notch starts (cf. Banchinae);

(5)  moderately long, regularly triangular in profile, medially membranous (cf. *Lycorina*);

(6) very elongate with lateral sides strongly converging towards the end, posterior margin angled with notch (cut) present and sometimes extending all the way to the base, in profile elongate and strongly triangular (cf. Acaenitinae);

(7) transverse with a cliff medially where ovipositor passes.

#222. **Pubescence of ovipositor sheath centrally ♀**

(0) with pubescence dense and short (longest hairs shorter than the breadth of ovipositor sheath);

(1) with pubescence dense and long (longest hairs exceeding the breadth of ovipositor sheath);

(2) with pubescence sparse and long (hairs separated by several times their diameter, and longer than breath of ovipositor sheath);

(3) with pubescence sparse and short;

(4) nearly hairless, only hairs are along the inner lower margin;

(5) nearly hairless, only some long sparse hairs on the apical half.

#223. **Shape of ovipositor sheaths** ♀

(0) more or less parallel sided, sometimes continuously enlarged towards apex or clearly enlarged at tip (cf. *Apechthis*);

(1) distinctly enlarged around middle, more narrow before and after (cf. *Thymaris*, *Zagryphus* in Tryphoninae).

#224. **Ovipositor length** [ordered] ♀

"Continuous character transformed to six states (0–5). Measured as the ratio of length of ovipositor sheaths to metasoma length.

#225. **Apex of ovipositor**

(0) more or less straight;

(1) up-curved;

(2) bowed downwards along at least posterior half;

(3) sinuous;

(4) abruptly decurved only close to the apex.

#226. **Base of ovipositor ventrally**

(0) simple;

(1) swollen (Figs 76, 77*).

#227. **Ovipositor in profile from base until the beginning of apex**

(0) parallel sided;

(1) evenly tapered;

(2) evenly tapered with enlarged submedian region of lower valve (Fig. 76*);

(3) lower valve widened twice, in front and after notch in dorsal valve (cf. Diplazontinae).

#228. **Ovipositor culpture on most of length**

(0) smooth, apparently unsculptured;

(1) matte;

(2) at least lower valve weakly to strongly wrinkled.

#229.  **Ovipositor transsection** [ordered]

(0) strongly compressed (Fig. 74*);

(1) subcylindrical, or weakly compressed (Fig. 73*);

(2) depressed.

#230. **Flexibility of apical region of ovipositro**

(0) rigid;

(1) capable of being deformed so extreme apex is deflected downwards.

#231. **Apex of lower valve of ovipositor**

(0) with distinctly oblique moderately interspaced teeth;

(1) without discernible teeth;

(2) with moderately interspaced teeth (except the most proximal one or two) vertical;

(3) with fine file-like teeth [outgroup only].

#232. **Lower valve just basal to apex**

"(0) without discrete scabrous areas;

1. with one or more small scabrous areas."

#233. **Lobe of lower valve**

"(0) not enclosing the upper (Figs 73, 74*);

1. with a clear lobe partially enclosing upper valve (Fig. 75*);
2. apically entirely enclosing the upper [outgroup only]."

#234. **Apex with basal tooth**

(0) unspecialized or absent;

(1) enlarged and forming barb.

#235. **Dorsal valve apically**

(0) smooth, simply tapered, with or without nodus;

(1) with lateral row of small denticles;

(2) with a dorsal row of low teeth;

(3) with dorsal, subapical notch (Fig.Wahl Fig. 4.01 *Exetastes*);

(4) slender and needle like (Wahl Fig. 1-33 *Astiphromma*);

(5) weakly sclerotized (cf. *Euceros*);

(6) slender, simply tapered (similar to 4 but polysphincini state);

(7) as state 3 but expanded laterally just before the notch;

(8) with two high teeth/nodi (cf. *Certonotus*);

(9) with two tubercules (cf. *Xorides*)."


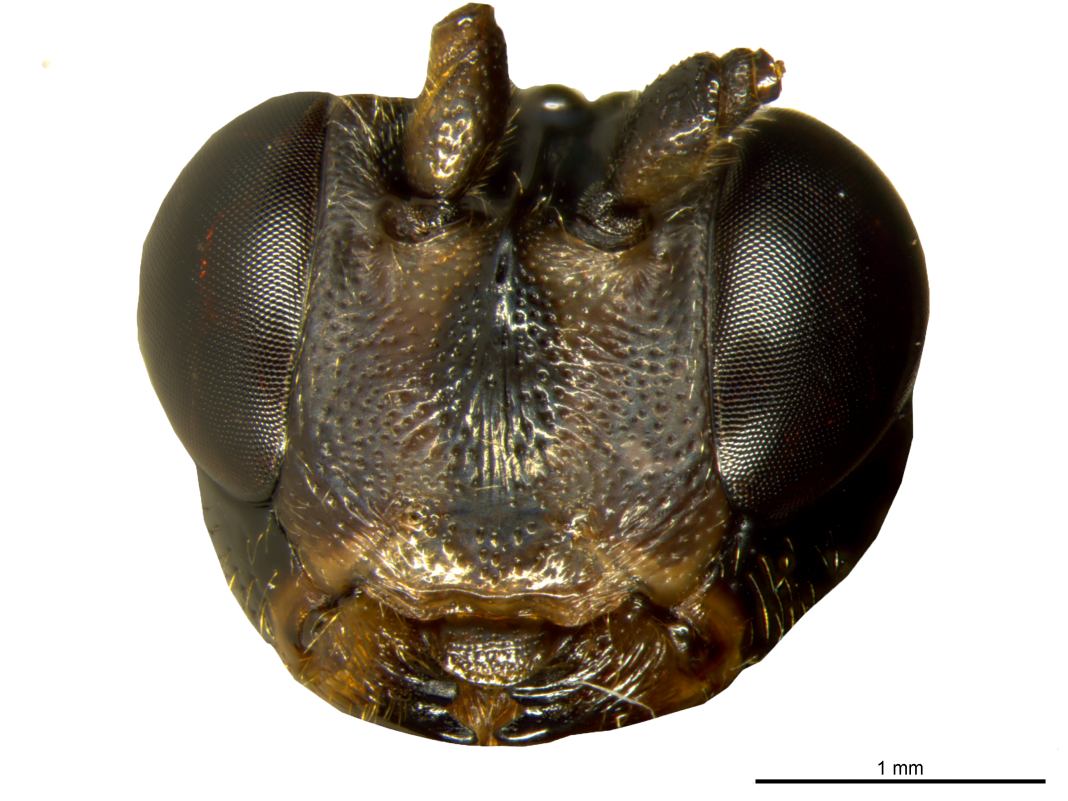


Figure 1. *Arotes moiwanus* (Acaenitini, Acaenitinae); head frontal view.


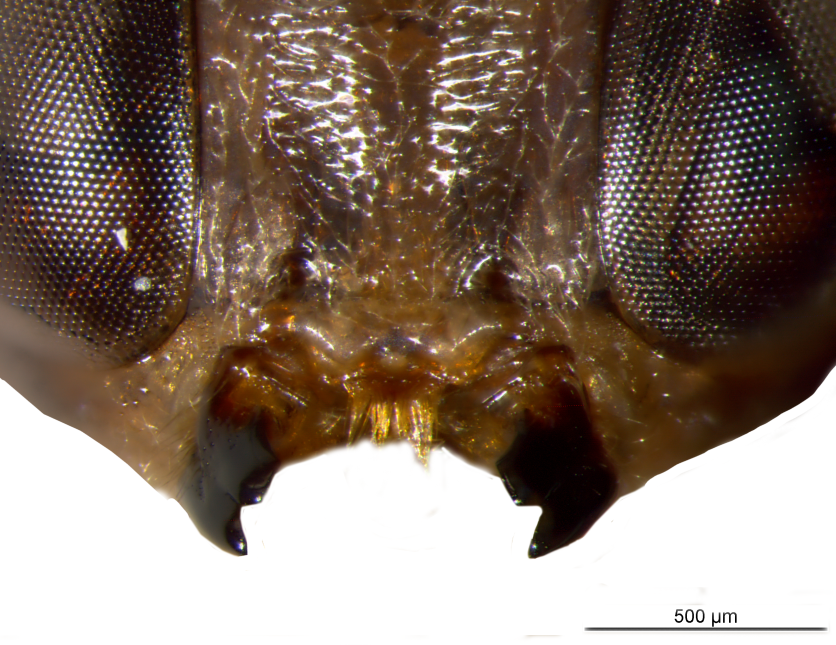


Figure 2. *Epirhyssa* sp. (Rhyssinae) ; head frontal view.


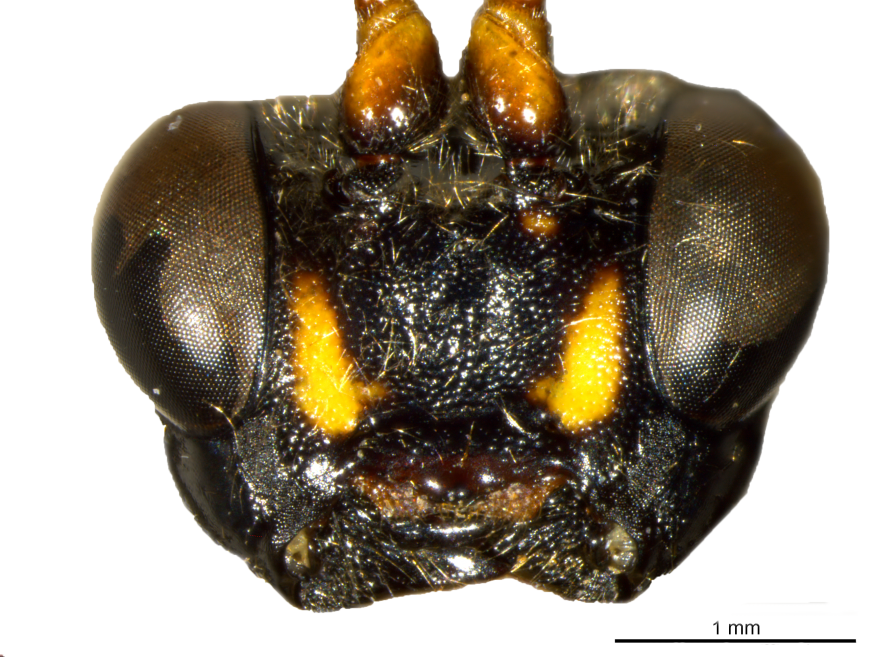


Figure 3. *Coleocentrus* sp. (Coleocentrini, Acaenitinae); head frontal view.


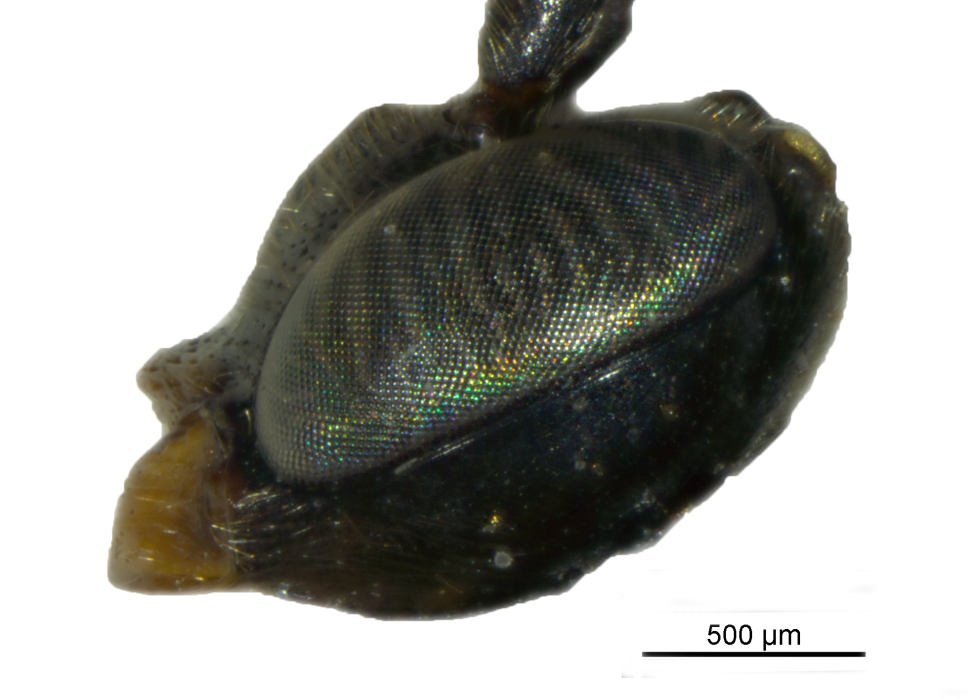


Eye height

Head height

Figure 4. *Xenoschesis* cf. *fulvicornis*; head lateral view.


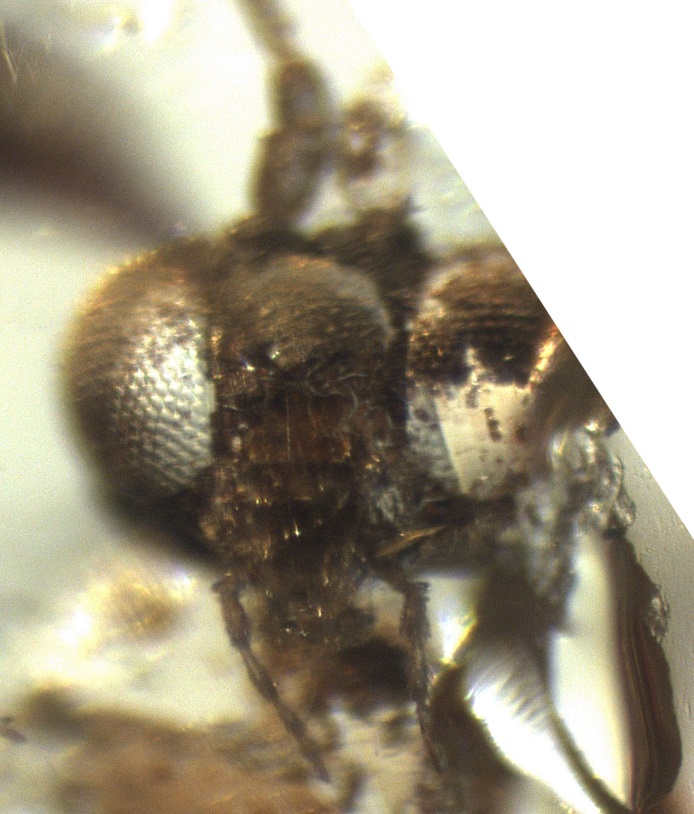


Figure 5. Cretaceous ichneumonid with transversely divided clypeus.


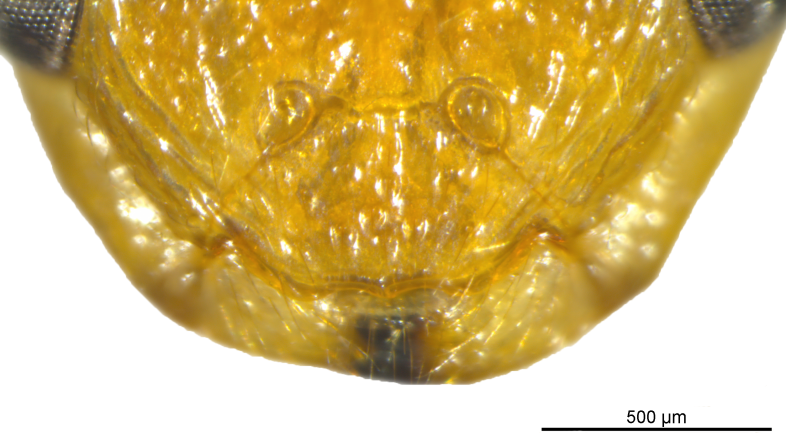


Figure 6. Trilobed apical margin of clypeus in *Phorotrophus congoensis* (#Ichn-2482)


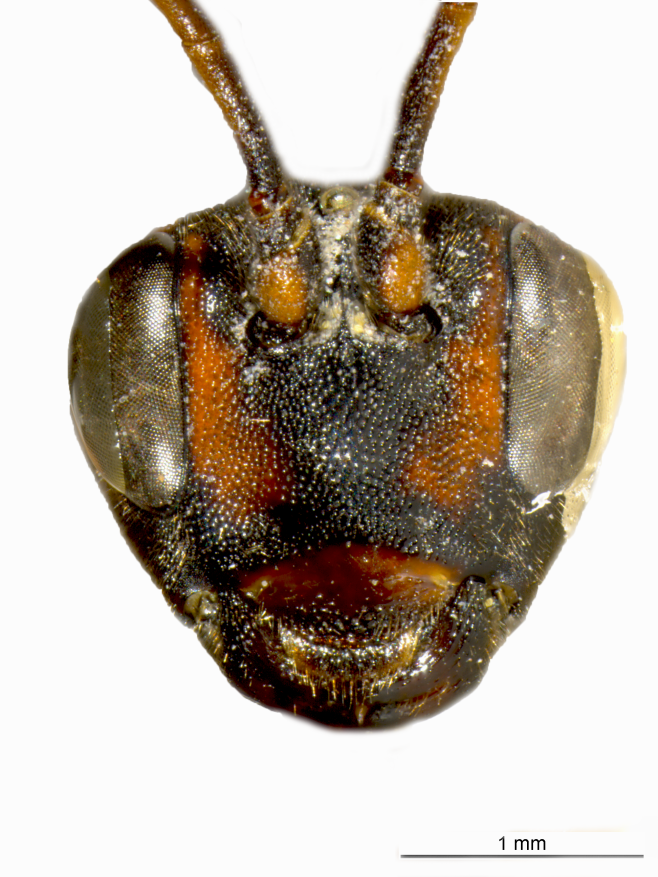


Figure 7. Strongly convex clypeus in *Procinetus decimator* (#Ichn-2536).


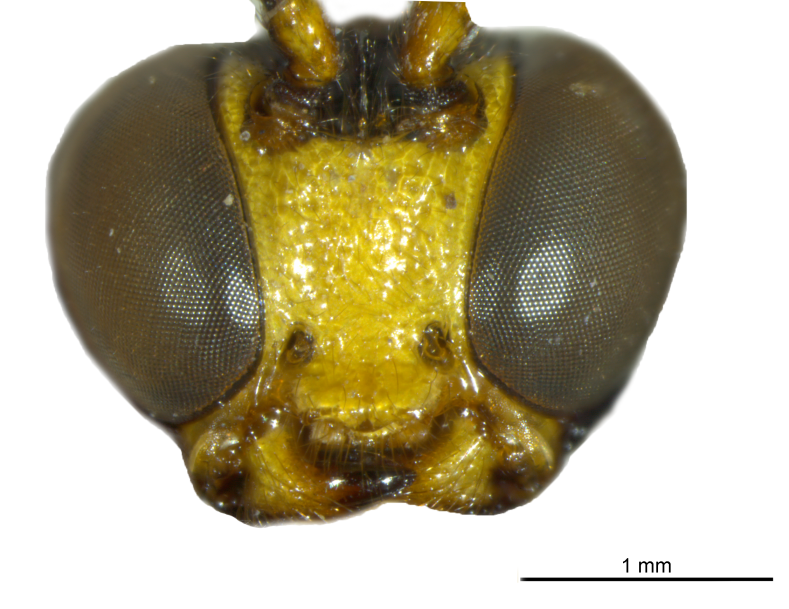


Figure 8. *Heteropelma amictum* (#Ichn-2531); head frontal view. Clypeus with two lateral, tooth-like protrusions. Mandible with the lower tooth almost completely twisted behind the upper tooth.


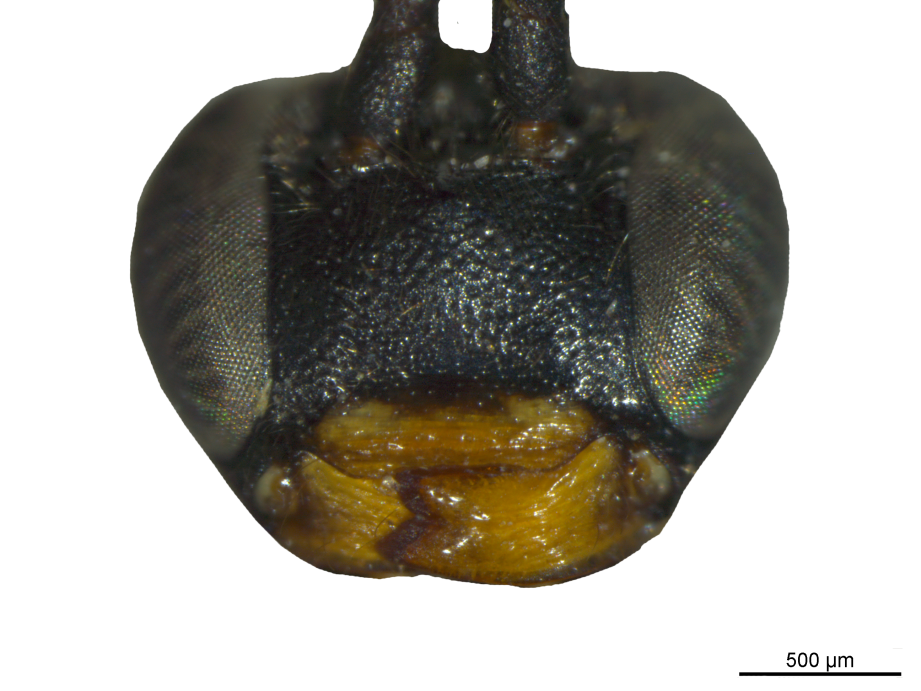


Figure 9. Xenoschesis cf. fulvicornis (#Ichn-2590); head frontal view. Clypeus laterally bilobed and broadly truncated centrally.


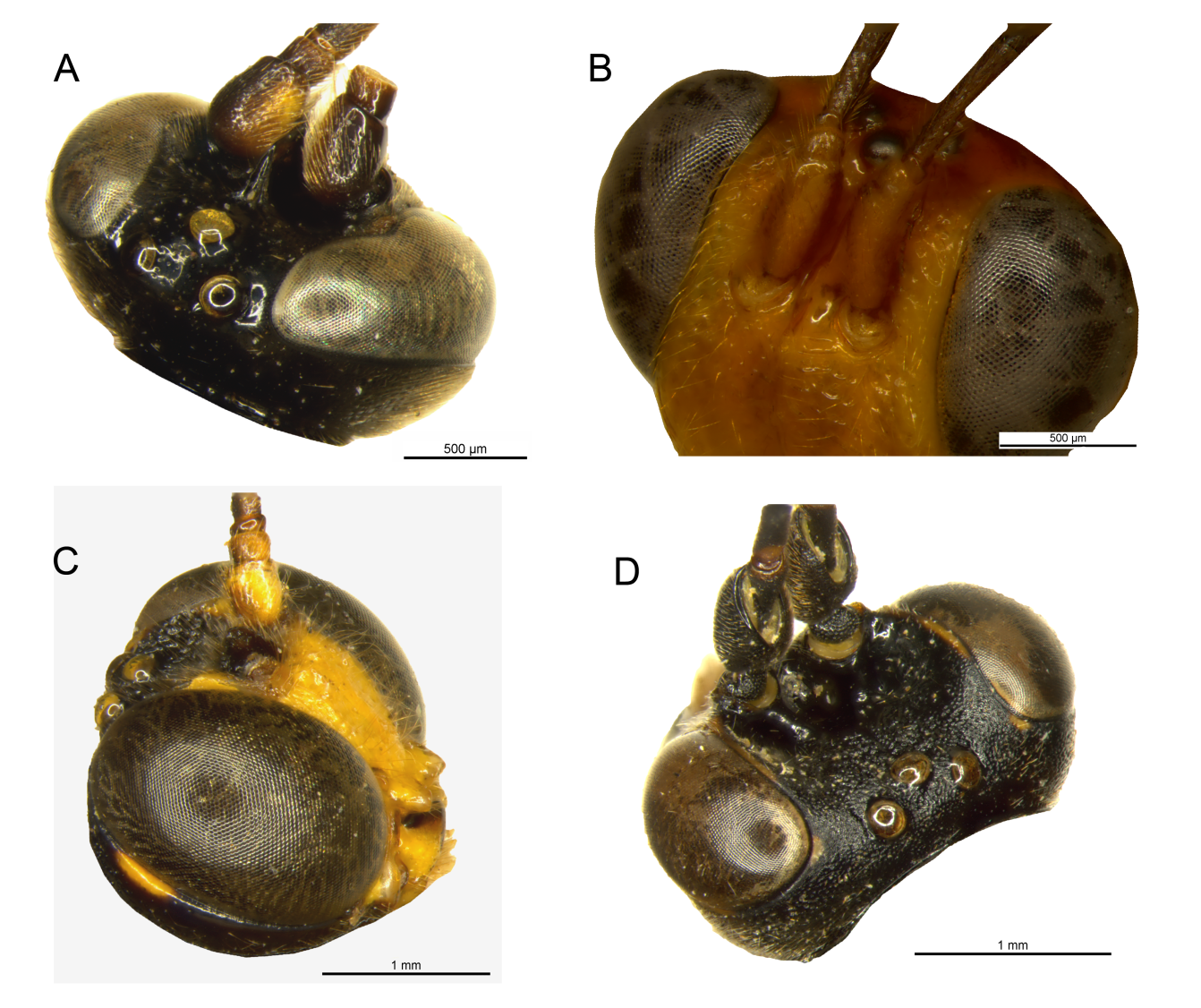


Figure 10. Modifications between antennal sockets and median ocellus. **A**, state (3) in *Colopotrochia*; **B**, state (5) in *Phorotrophus*; **C**, state (6) in *Heteropelma*; **D**, state (7) in *Buathra*.

**Carinae**

L – median longitudinal carina

K – lateral longitudinal carina

J – pleural carina

M – anterior transverse carina

N – posterior transverse carina

O – apophysis or crest

H – juxacoxal carina

**Areas**

30 – basal area

31 – areola or area superomedia

32 – petiolar area

23 – first lateral area

24 – second lateral area

25 – third lateral area

26 – first pleural area

27 – second pleural area

28 – third pleural area

29 – spiracle

15 – metapleurum

16 – juxacoxal area

according to Townes, 1981

Figure 11. Propodeal carination adapted from Townes (1969).

******

Fig 12. Example of variation in propodeal carination in diplazontine (from Klopfstein 2014).


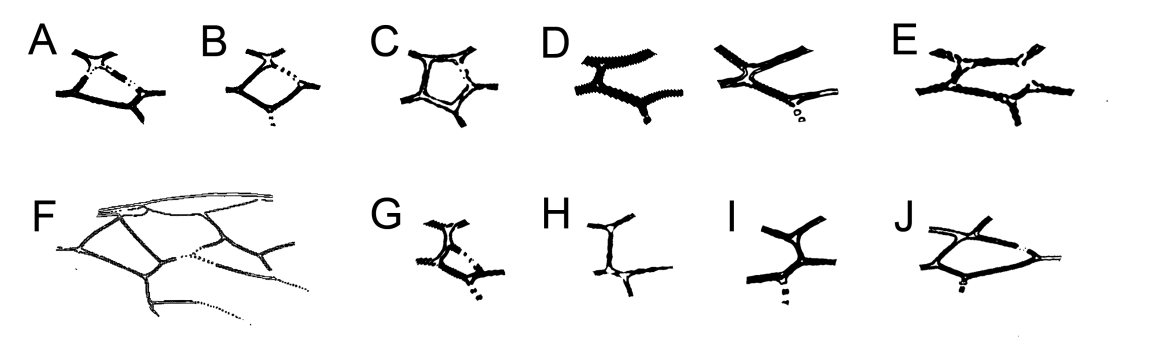


Fig 13. Illustration of coded areolet shapes A–J (areolets modified from Goulet & Hubert 1993; Kopylov 2010; Townes 1969a, 1969b, 1971).
