# Supplementary material for: Illustrating phylogenetic placement of fossils using RoguePlots: An example from ichneumonid parasitoid wasps (Hymenoptera, Ichneumonidae) and an extensive morphological matrix
Source: PLoS One. 2019 Apr 2;14(4):e0212942. doi: 10.1371/journal.pone.0212942 (PMC6445432; doi:10.1371/journal.pone.0212942)

Pim_Pimpla_eocenica

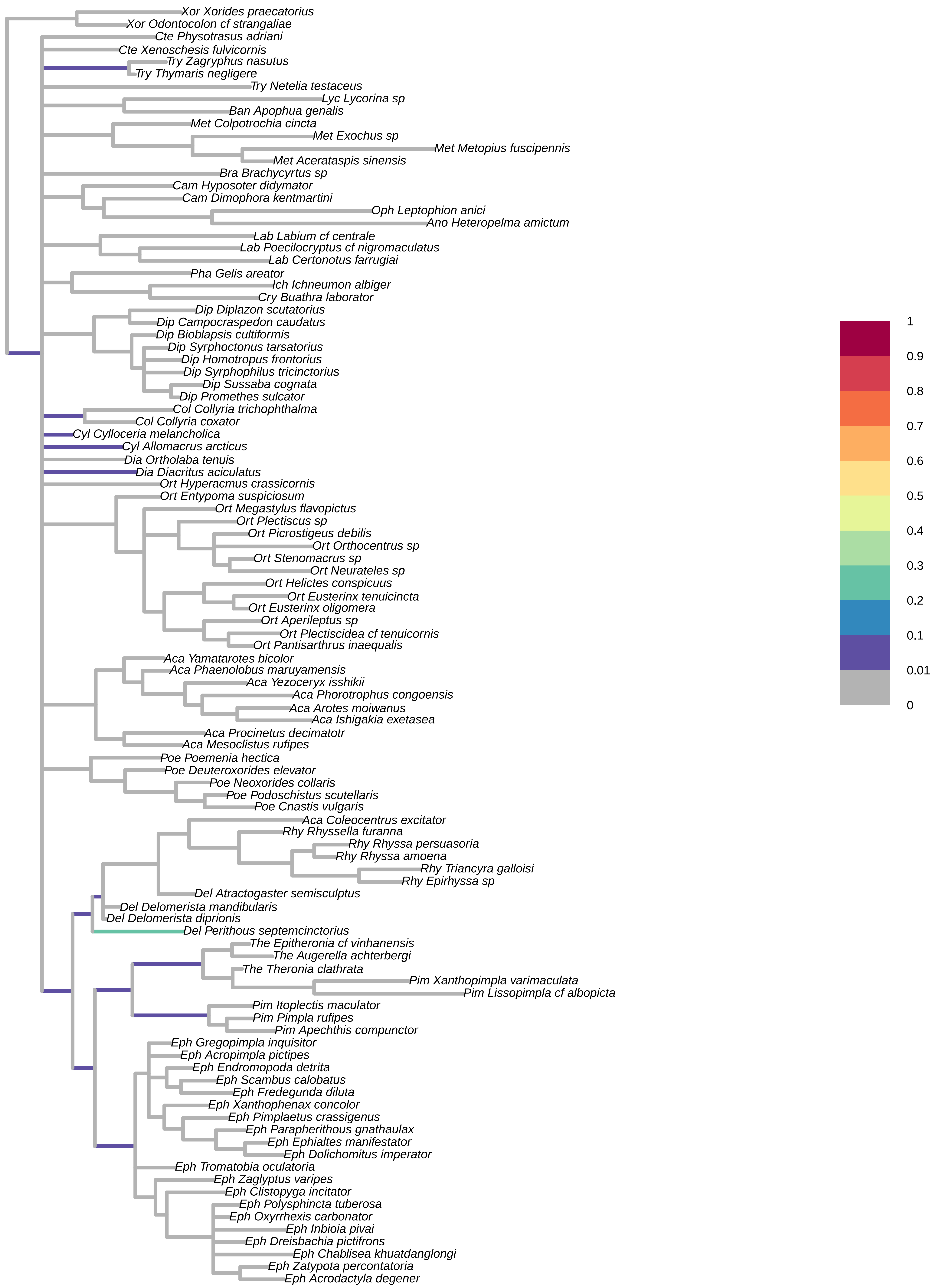

ICH_Eopimpla_grandis

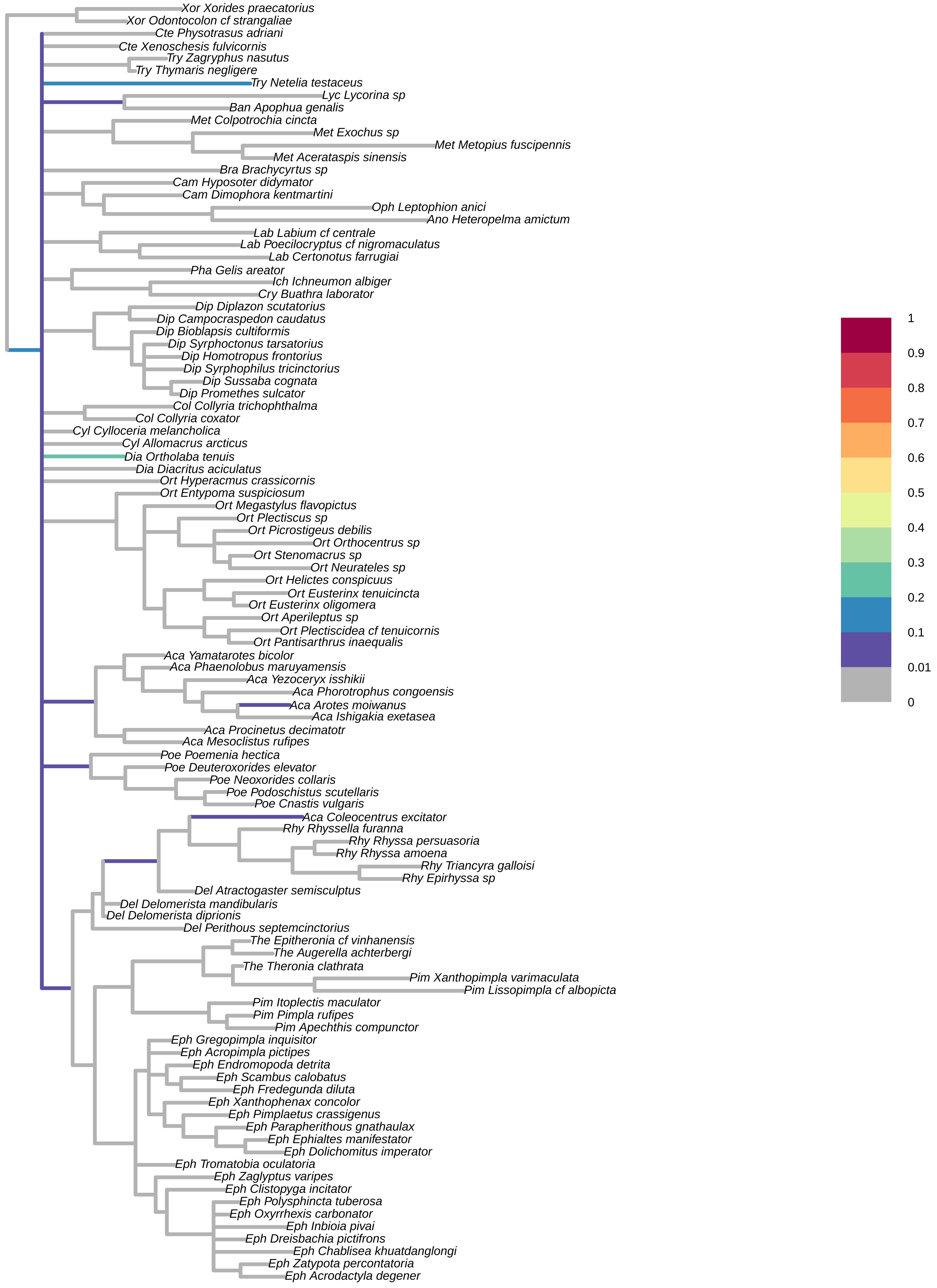

ICH_Ichninsum_appendicrassus

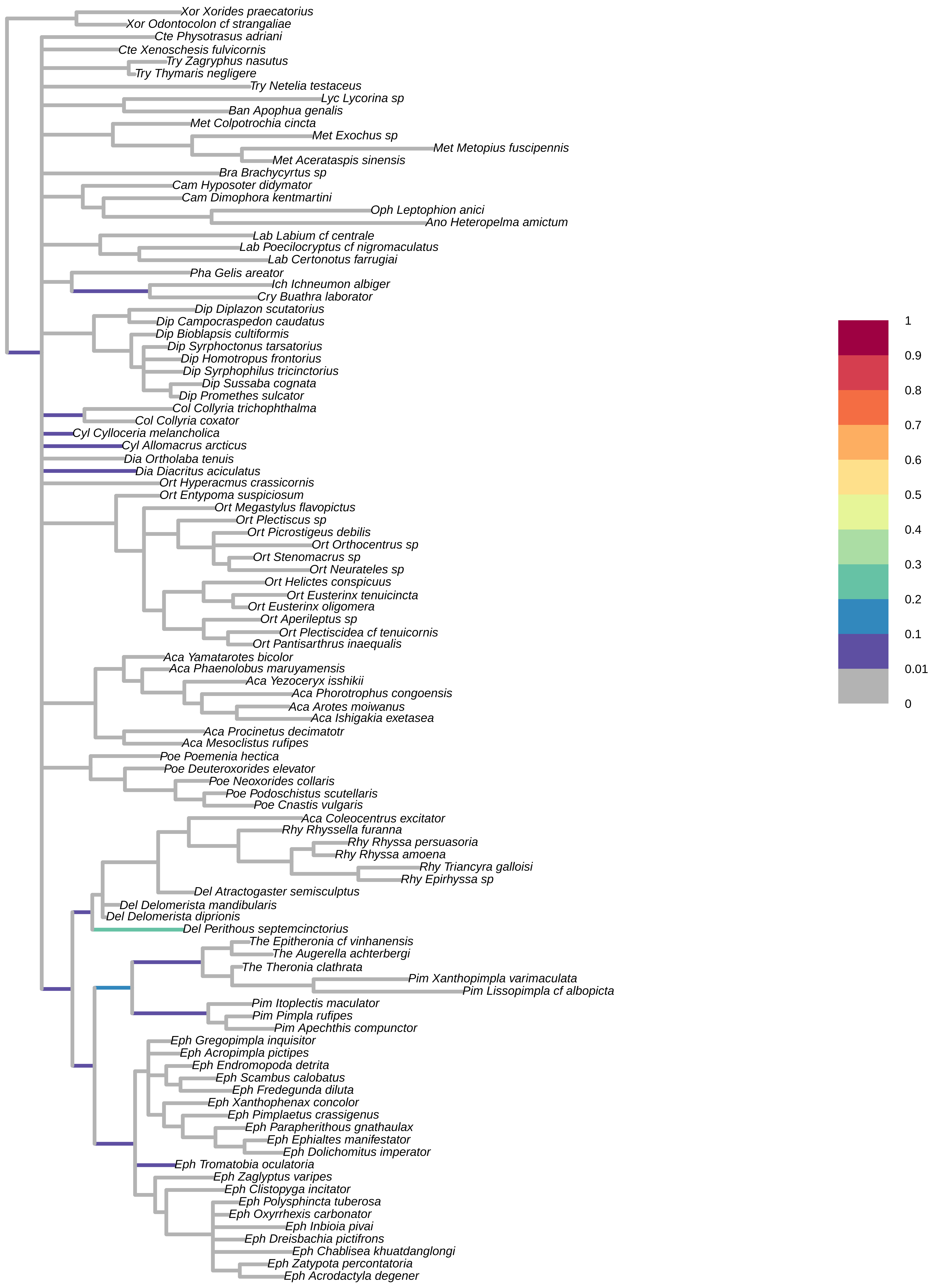

Aca_Mesoclistus_yamataroti

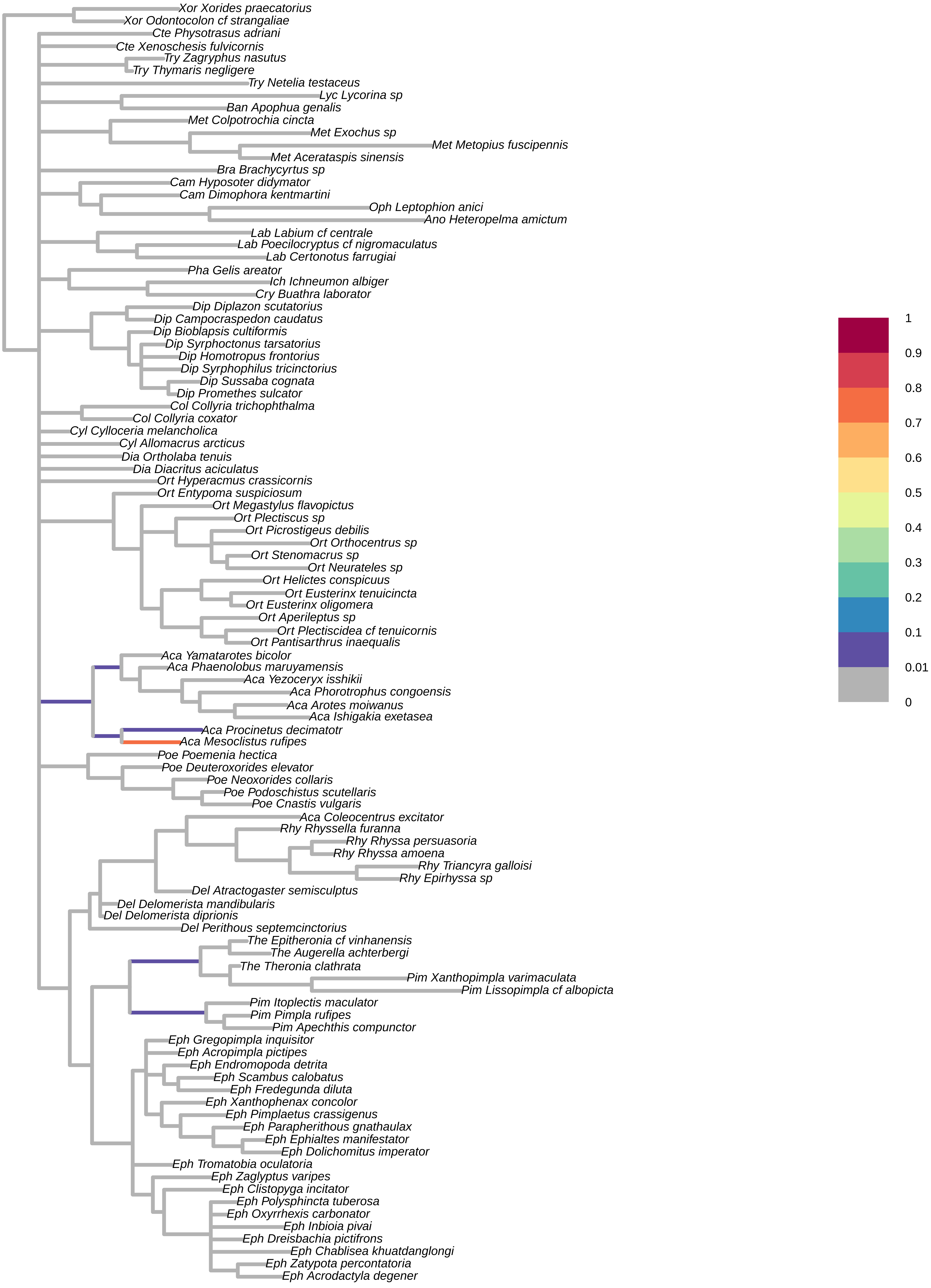

ICH_Lithotorus_cressoni

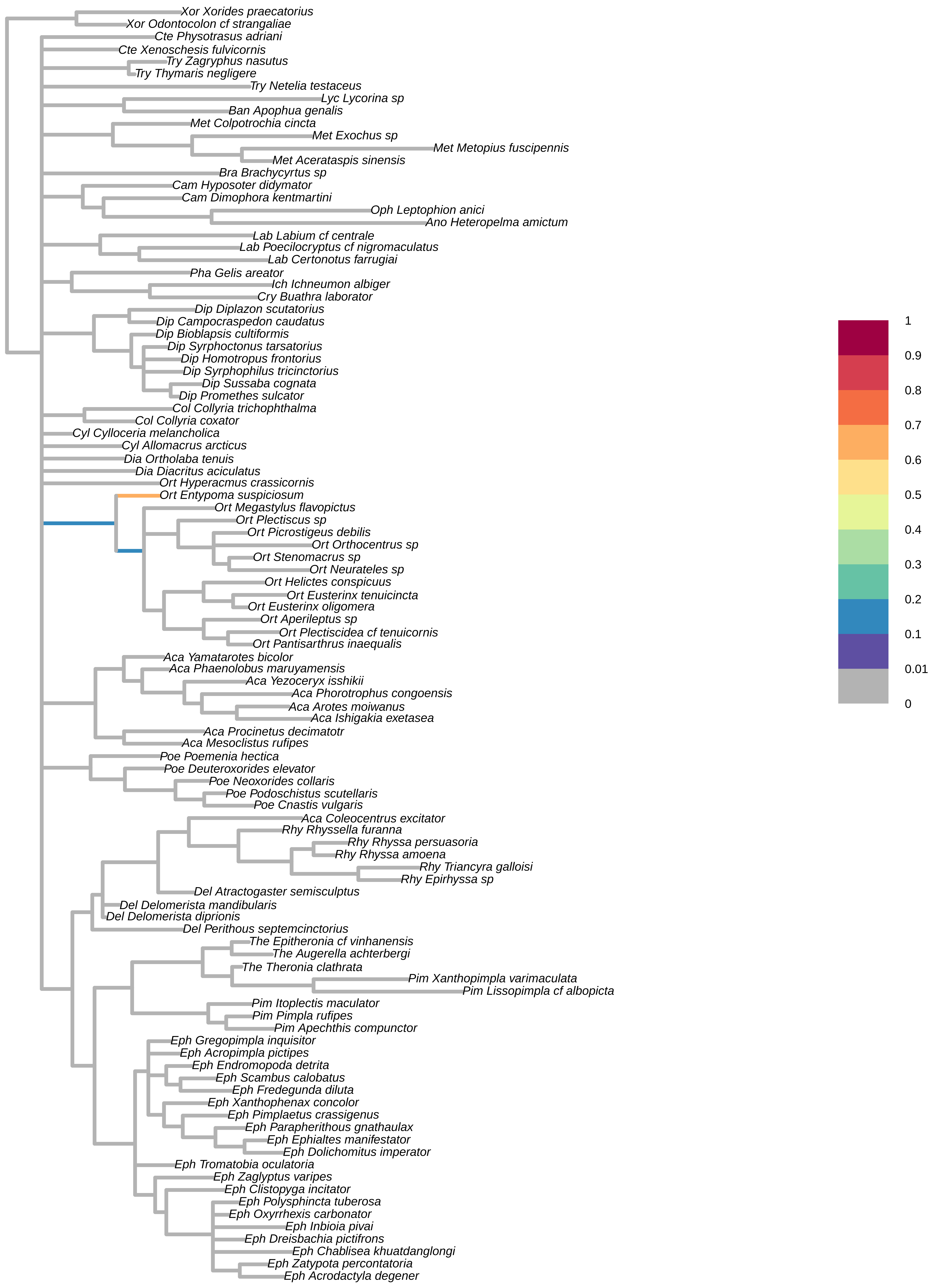

PIM_Crusopimpla_rediviva

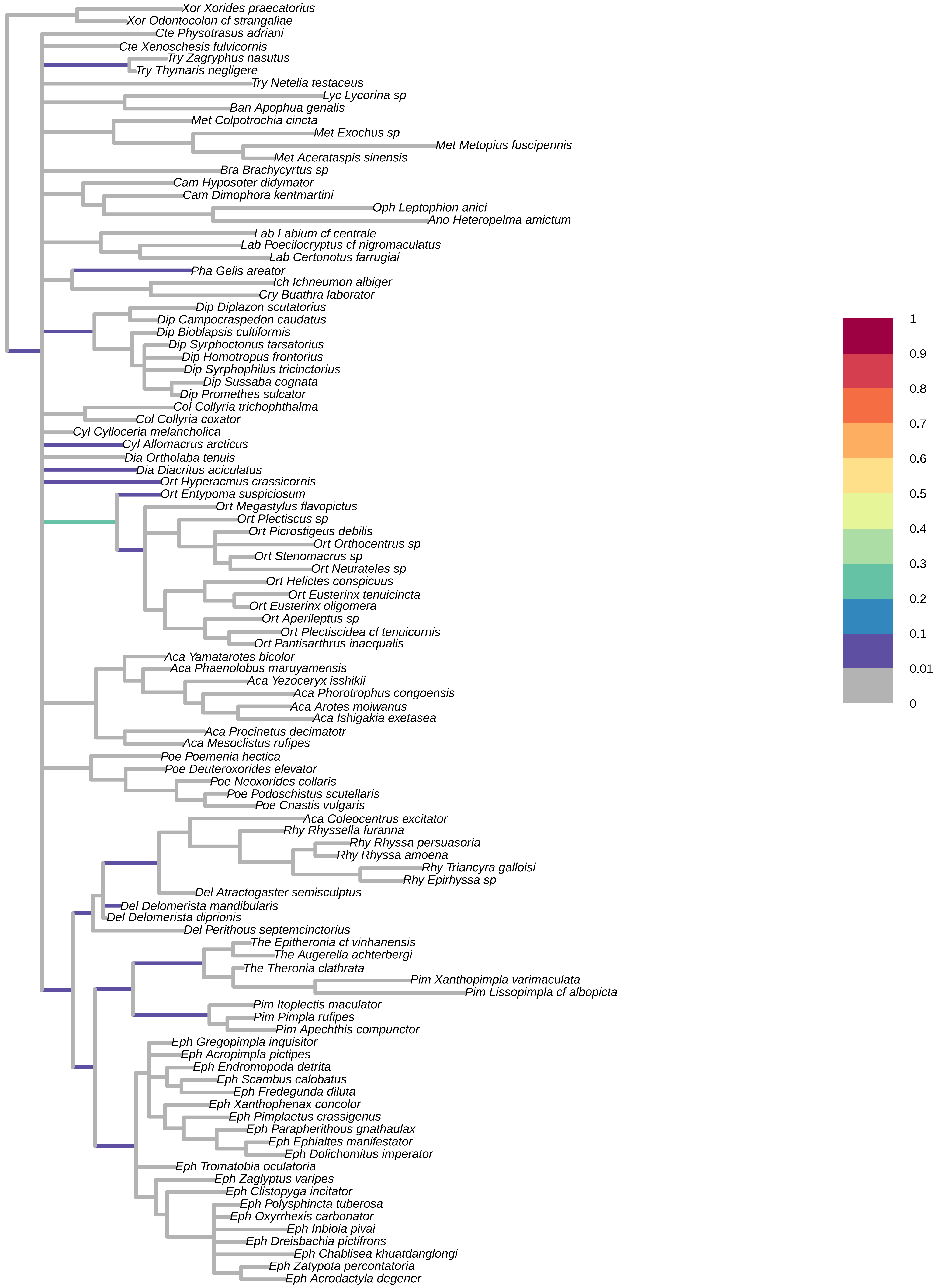

Eph_Dolichomitus_saxeus

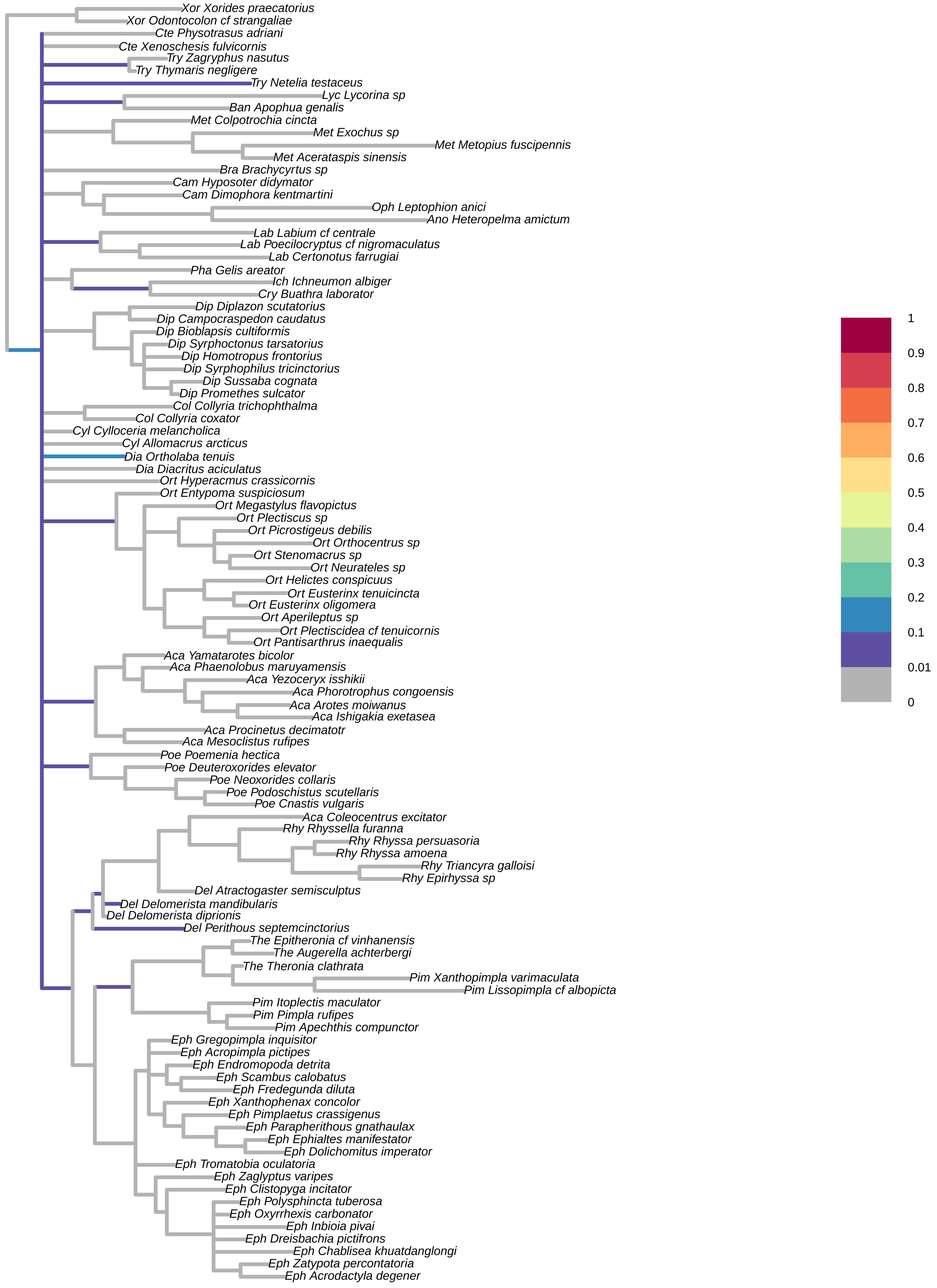

Ban_Glypta_transversalis

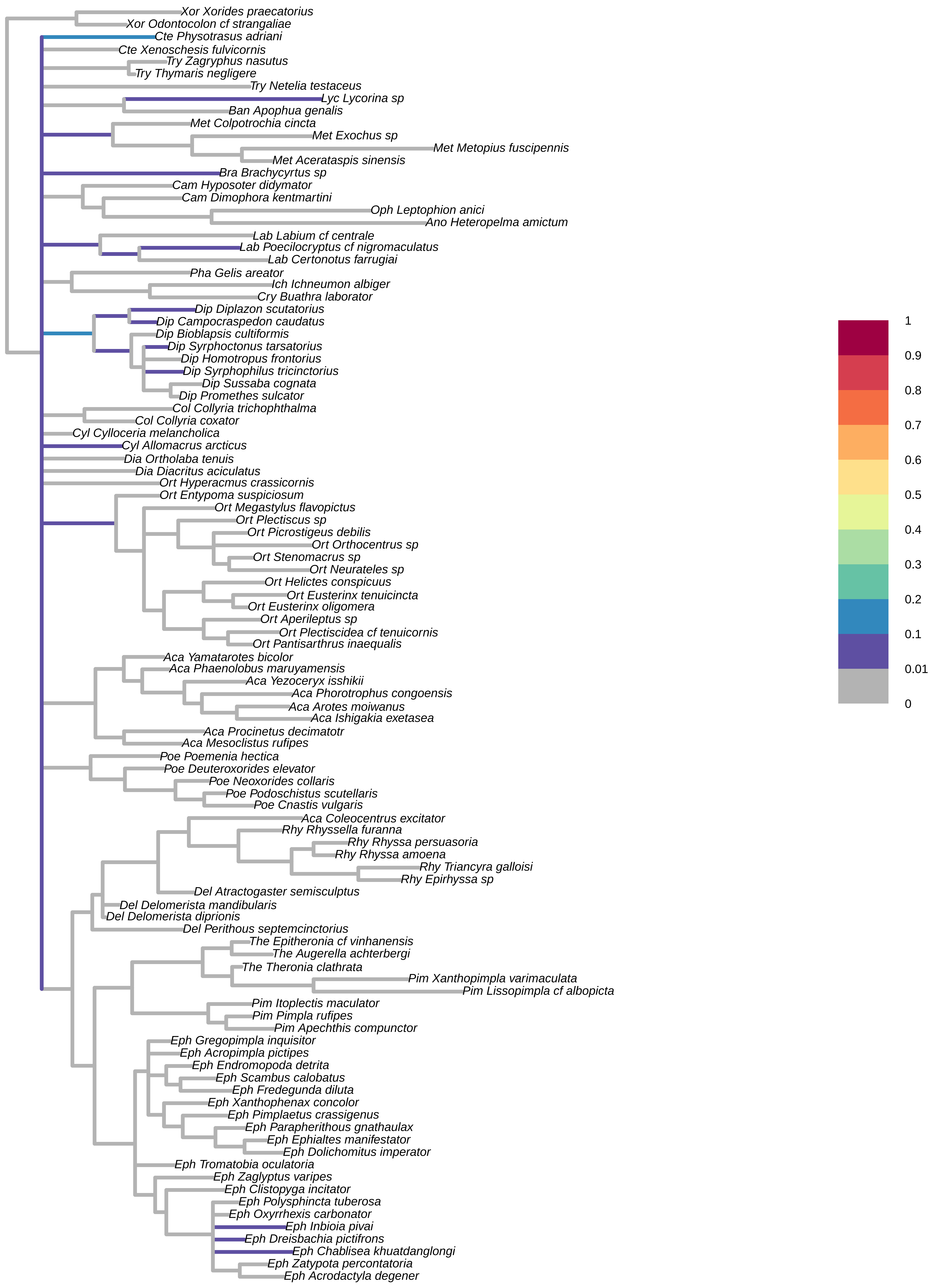

PIM_Crusopimpla_tadushiensis

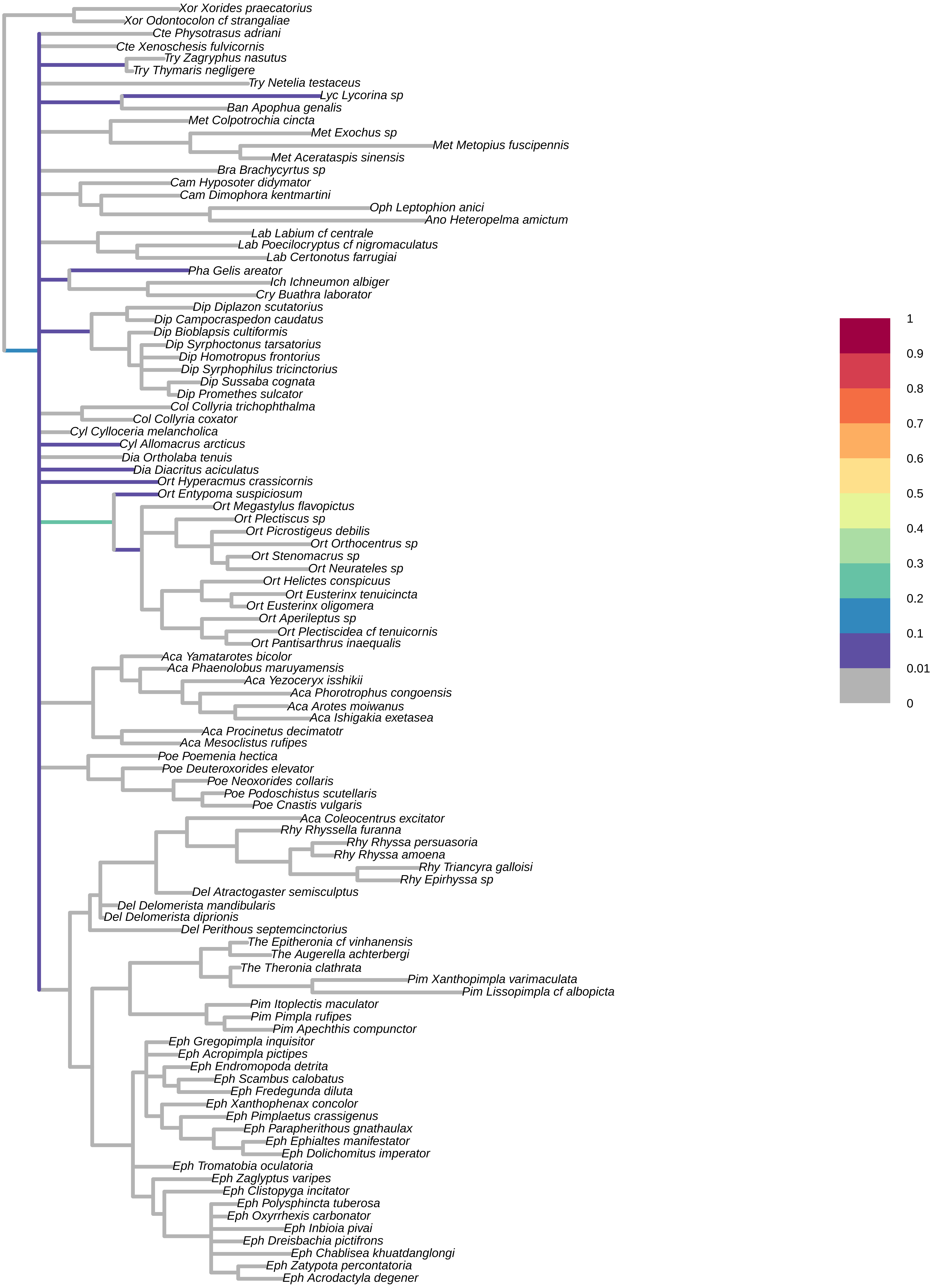

ICH_Tilgidopsis_heaesitans

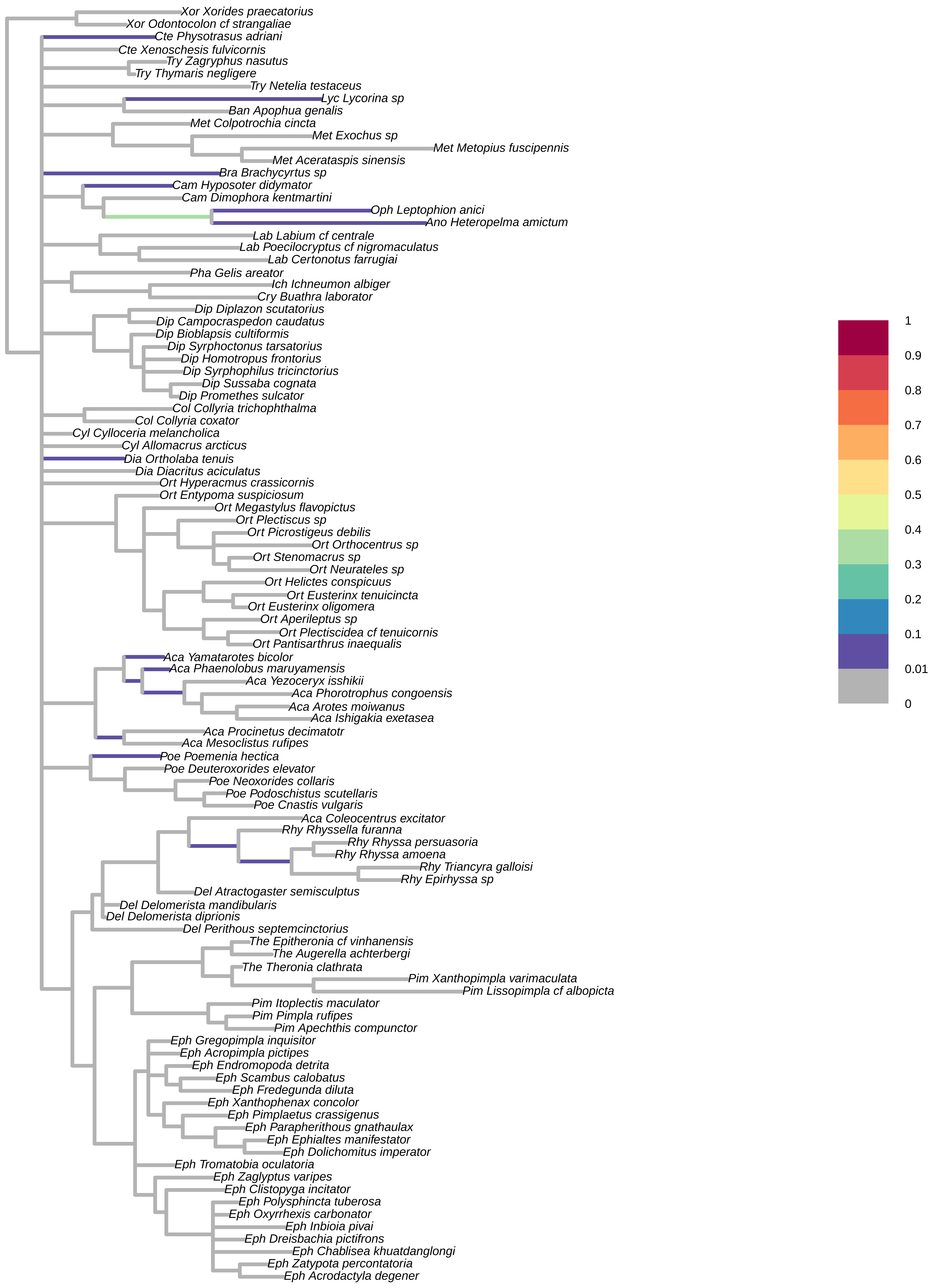

Cry_Phygadeuon_petrifactellus

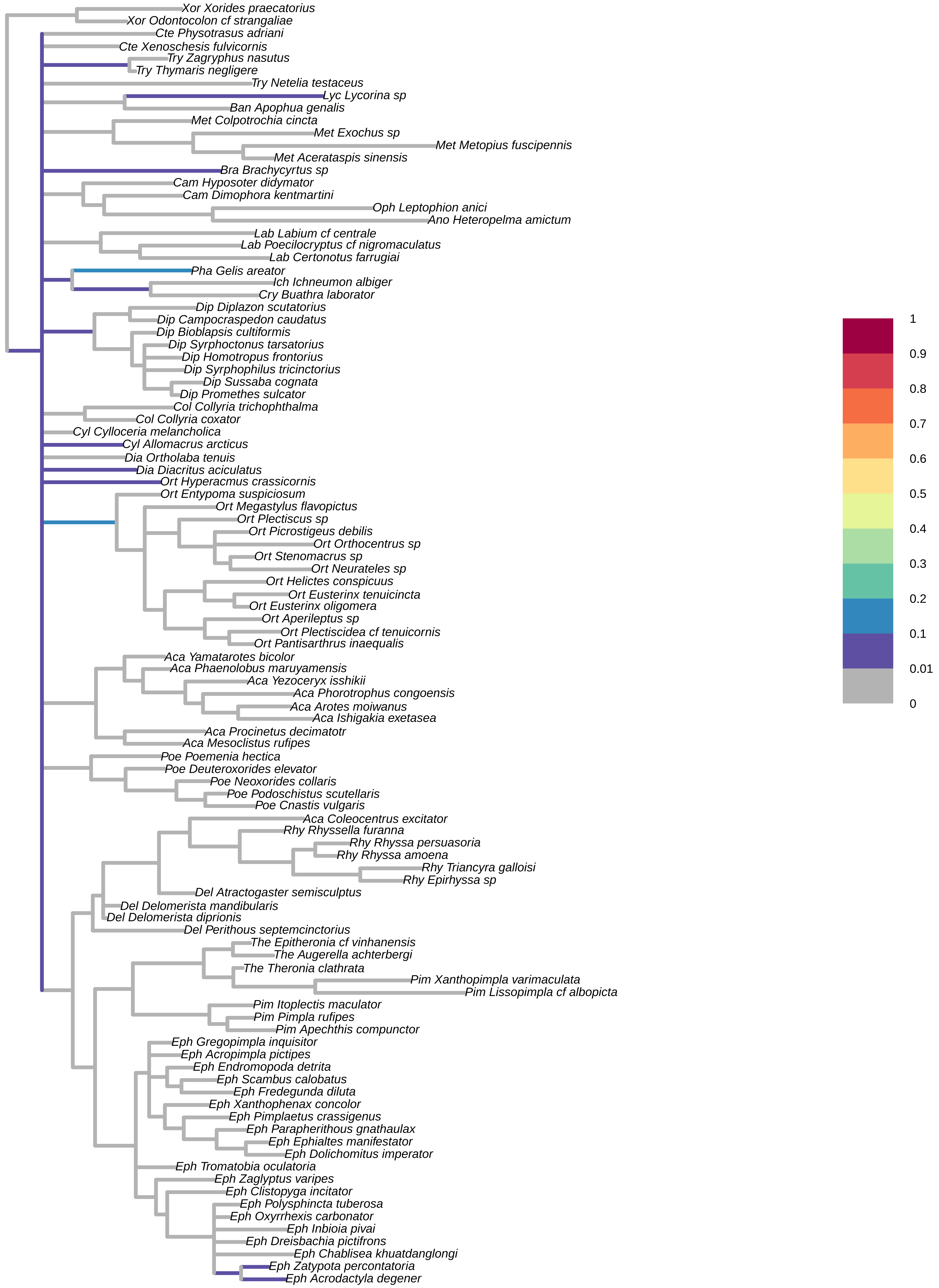

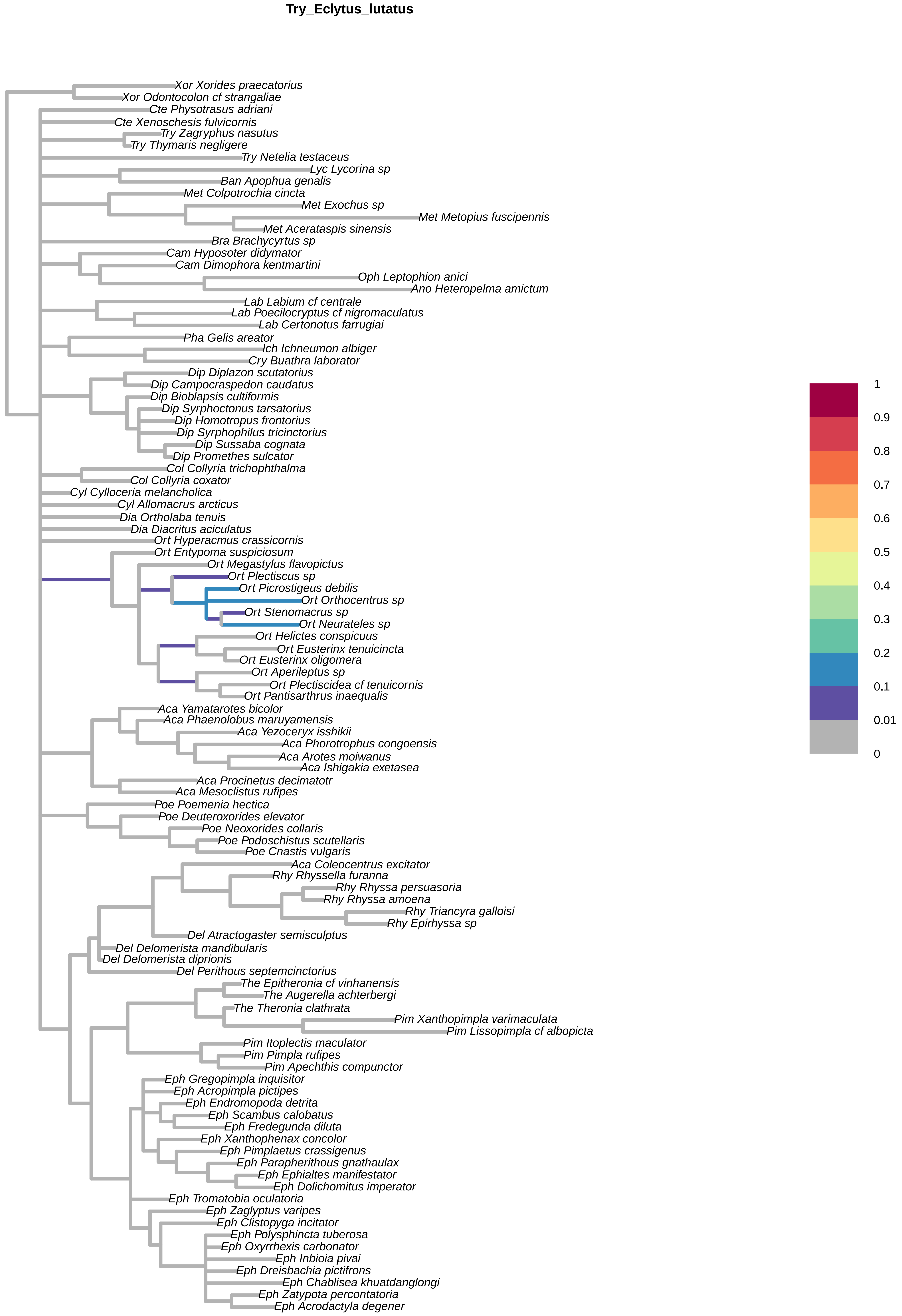
Try_Eclytus_lutatus
Xor Xorides praecatorius
Xor Odontocolon cf strangaliae
Cte Physotrasus adriani
Cte Xenoschesis fulvicornis
Try Zagryphus nasutus
Try Thymaris negligere
Try Netelia testaceus
Lyc Lycorina sp
Ban Apophua genalis
Met Colpotrochia cincta
Met Exochus sp
Met Metopius fuscipennis
Met Acerataspis sinensis
Bra Brachycyrtus sp
Cam Hyposoter didymator
Cam Dimophora kentmartini
Oph Leptophion anici
Ano Heteropelma amictum
Lab Labium cf centrale
Lab Poecilocryptus cf nigromaculatus
Lab Certonotus farrugiai
Pha Gelis areator
Ich Ichneumon albiger
Cry Buathra laborator
Dip Diplazon scutatorius
Dip Campocraspedon caudatus
Dip Bioblapsis cultiformis
Dip Syrphoctonus tarsatorius
Dip Homotropus frontorius
Dip Syrphophilus tricinctorius
Dip Sussaba cognata
Dip Promethes sulcator
Col Collyria trichophthalma
Col Collyria coxator
Cyl Cylloceria melancholica
Cyl Allomacrus arcticus
Dia Ortholaba tenuis
Dia Diacritus aciculatus
Ort Hyperacmus crassicornis
Ort Entypoma suspiciosum
Ort Megastylus flavopictus
Ort Plectiscus sp
Ort Picrostigeus debilis
Ort Orthocentrus sp
Ort Stenomacrus sp
Ort Neurateles sp
Ort Helictes conspicuus
Ort Eusterinx tenuicincta
Ort Eusterinx oligomera
Ort Aperileptus sp
Ort Plectiscidea cf tenuicornis
Ort Pantisarthrus inaequalis
Aca Yamatarotes bicolor
Aca Phaenolobus maruyamensis
Aca Yezoceryx isshikii
Aca Phorotrophus congoensis
Aca Arotes moiwanus
Aca Ishigakia exetasea
Aca Procinetus decimatotr
Aca Mesoclistus rufipes
Poe Poemenia hectica
Poe Deuteroxorides elevator
Poe Neoxorides collaris
Poe Podoschistus scutellaris
Poe Cnastis vulgaris
Aca Coleocentrus excitator
Rhy Rhyssella furanna
Rhy Rhyssa persuasoria
Rhy Rhyssa amoena
Rhy Triancyra galloisi
Rhy Epirhyssa sp
Del Atractogaster semisculptus
Del Delomerista mandibularis
Del Delomerista diprionis
Del Perithous septemcinctorius
The Epitheronia cf vinhanensis
The Augerella achterbergi
The Theronia clathrata
Pim Xanthopimpla varimaculata
Pim Lissopimpla cf albopicta
Pim Itoplectis maculator
Pim Pimpla rufipes
Pim Apechthis compunctor
Eph Gregopimpla inquisitor
Eph Acropimpla pictipes
Eph Endromopoda detrita
Eph Scambus calobatus
Eph Fredegunda diluta
Eph Xanthophenax concolor
Eph Pimplaetus crassigenus
Eph Parapherithous gnathaulax
Eph Ephialtes manifestator
Eph Dolichomitus imperator
Eph Tromatobia oculatoria
Eph Zaglyptus varipes
Eph Clistopyga incitator
Eph Polysphincta tuberosa
Eph Oxyrrhexis carbonator
Eph Inbioia pivai
Eph Dreisbachia pictifrons
Eph Chablisea khuatdanglongi
Eph Zatypota percontatoria
Eph Acrodactyla degener
1
0.9
0.8
0.7
0.6
0.5
0.4
0.3
0.2
0.1
0.01
0

ICH_Trymectus_amasidis

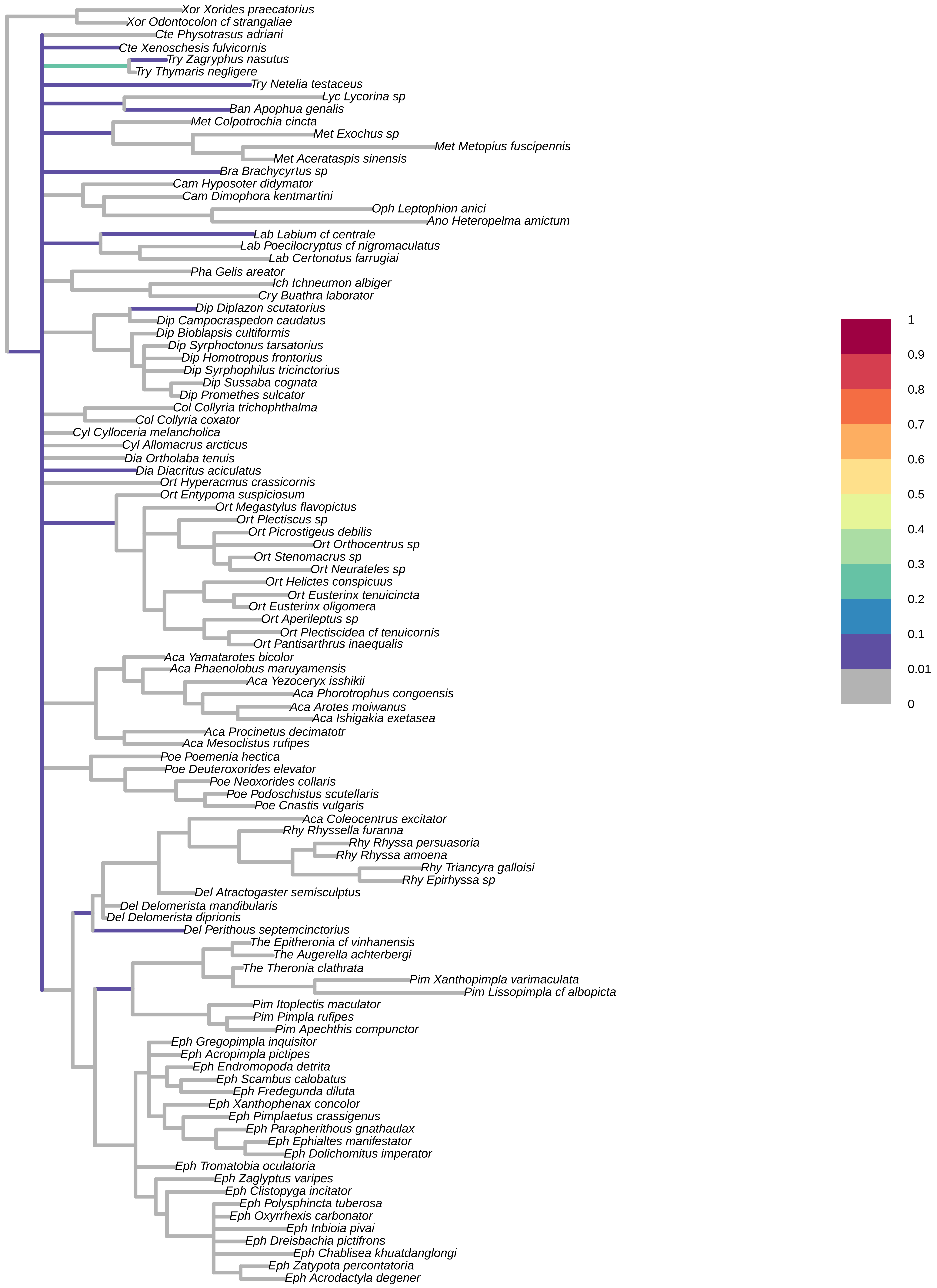

Ort_Plectiscidea_lanhami

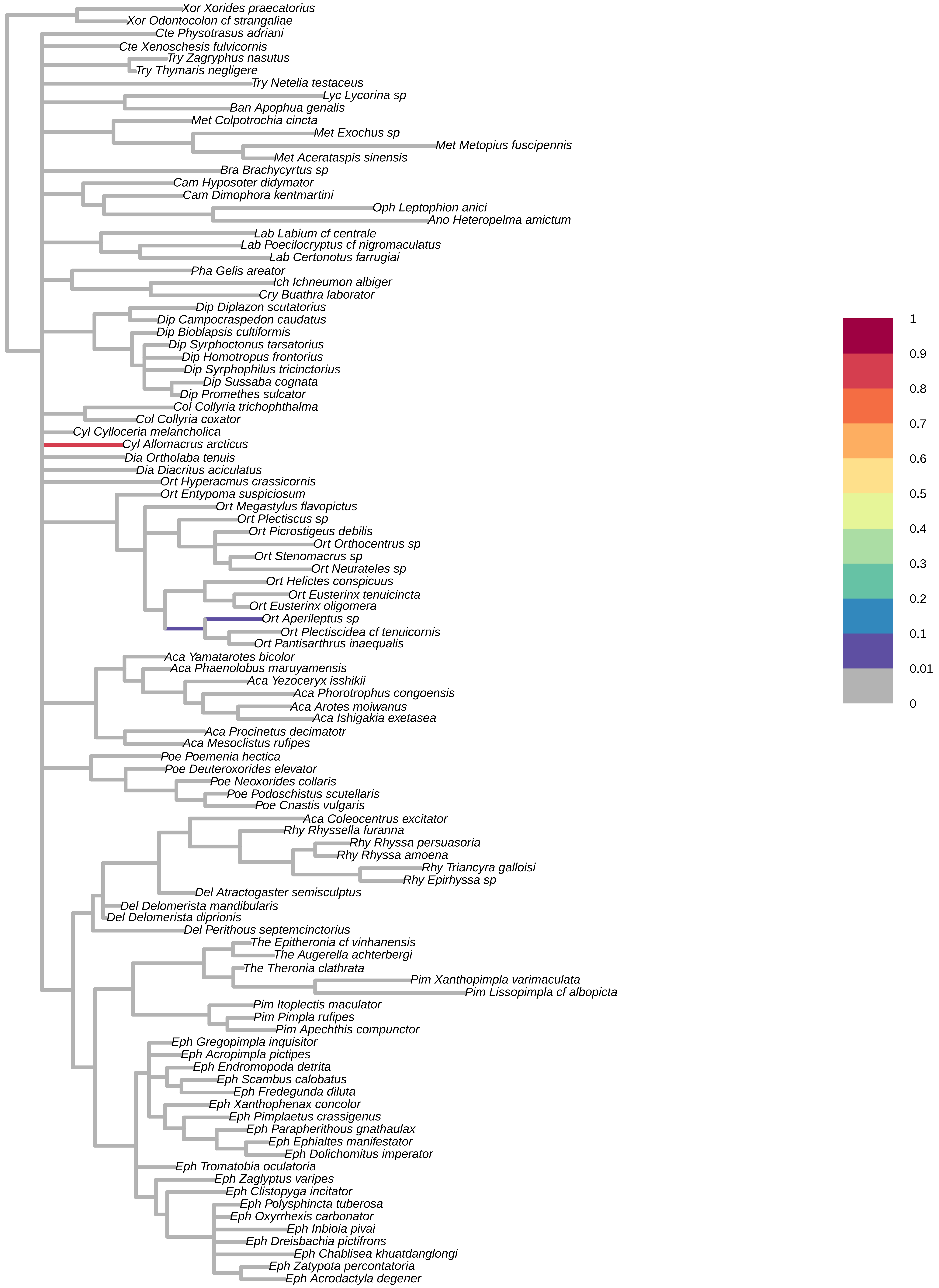

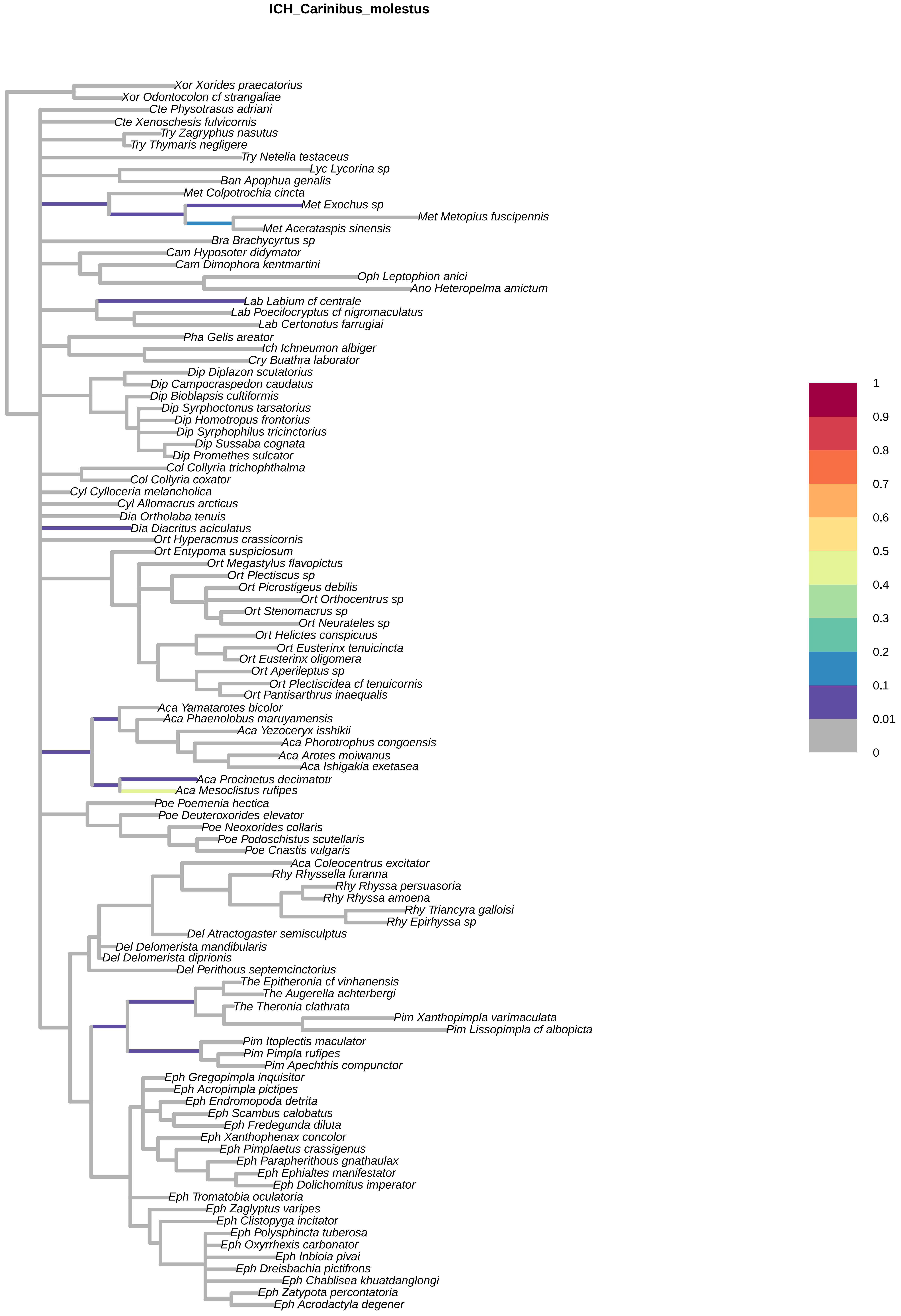
ICH_Carinibus_molestus
Xor Xorides praecatorius
Xor Odontocolon cf strangaliae
Cte Physotrasus adriani
Cte Xenoschesis fulvicornis
Try Zagryphus nasutus
Try Thymaris negligere
Try Netelia testaceus
Lyc Lycorina sp
Ban Apophua genalis
Met Colpotrochia cincta
Met Exochus sp
Met Metopius fuscipennis
Met Acerataspis sinensis
Bra Brachycyrtus sp
Cam Hyposoter didymator
Cam Dimophora kentmartini
Oph Leptophion anici
Ano Heteropelma amictum
Lab Labium cf centrale
Lab Poecilocryptus cf nigromaculatus
Lab Certonotus farrugiai
Pha Gelis areator
Ich Ichneumon albiger
Cry Buathra laborator
Dip Diplazon scutatorius
Dip Campocraspedon caudatus
Dip Bioblapsis cultiformis
Dip Syrphoctonus tarsatorius
Dip Homotropus frontorius
Dip Syrphophilus tricinctorius
Dip Sussaba cognata
Dip Promethes sulcator
Col Collyria trichophthalma
Col Collyria coxator
Cyl Cylloceria melancholica
Cyl Allomacrus arcticus
Dia Ortholaba tenuis
Dia Diacritus aciculatus
Ort Hyperacmus crassicornis
Ort Entypoma suspiciosum
Ort Megastylus flavopictus
Ort Plectiscus sp
Ort Picrostigeus debilis
Ort Orthocentrus sp
Ort Stenomacrus sp
Ort Neurateles sp
Ort Helictes conspicuus
Ort Eusterinx tenuicincta
Ort Eusterinx oligomera
Ort Aperileptus sp
Ort Plectiscidea cf tenuicornis
Ort Pantisarthrus inaequalis
Aca Yamatarotes bicolor
Aca Phaenolobus maruyamensis
Aca Yezoceryx isshikii
Aca Phorotrophus congoensis
Aca Arotes moiwanus
Aca Ishigakia exetasea
Aca Procinetus decimatotr
Aca Mesoclistus rufipes
Poe Poemenia hectica
Poe Deuteroxorides elevator
Poe Neoxorides collaris
Poe Podoschistus scutellaris
Poe Cnastis vulgaris
Aca Coleocentrus excitator
Rhy Rhyssella furanna
Rhy Rhyssa persuasoria
Rhy Rhyssa amoena
Rhy Triancyra galloisi
Rhy Epirhyssa sp
Del Atractogaster semisculptus
Del Delomerista mandibularis
Del Delomerista diprionis
Del Perithous septemcinctorius
The Epitheronia cf vinhanensis
The Augerella achterbergi
The Theronia clathrata
Pim Xanthopimpla varimaculata
Pim Lissopimpla cf albopicta
Pim Itoplectis maculator
Pim Pimpla rufipes
Pim Apechthis compunctor
Eph Gregopimpla inquisitor
Eph Acropimpla pictipes
Eph Endromopoda detrita
Eph Scambus calobatus
Eph Fredegunda diluta
Eph Xanthophenax concolor
Eph Pimplaetus crassigenus
Eph Parapherithous gnathaulax
Eph Ephialtes manifestator
Eph Dolichomitus imperator
Eph Tromatobia oculatoria
Eph Zaglyptus varipes
Eph Clistopyga incitator
Eph Polysphincta tuberosa
Eph Oxyrrhexis carbonator
Eph Inbioia pivai
Eph Dreisbachia pictifrons
Eph Chablisea khuatdanglongi
Eph Zatypota percontatoria
Eph Acrodactyla degener
1
0.9
0.8
0.7
0.6
0.5
0.4
0.3
0.2
0.1
0.01
0

PIM_Xanthopimpla_messelensis

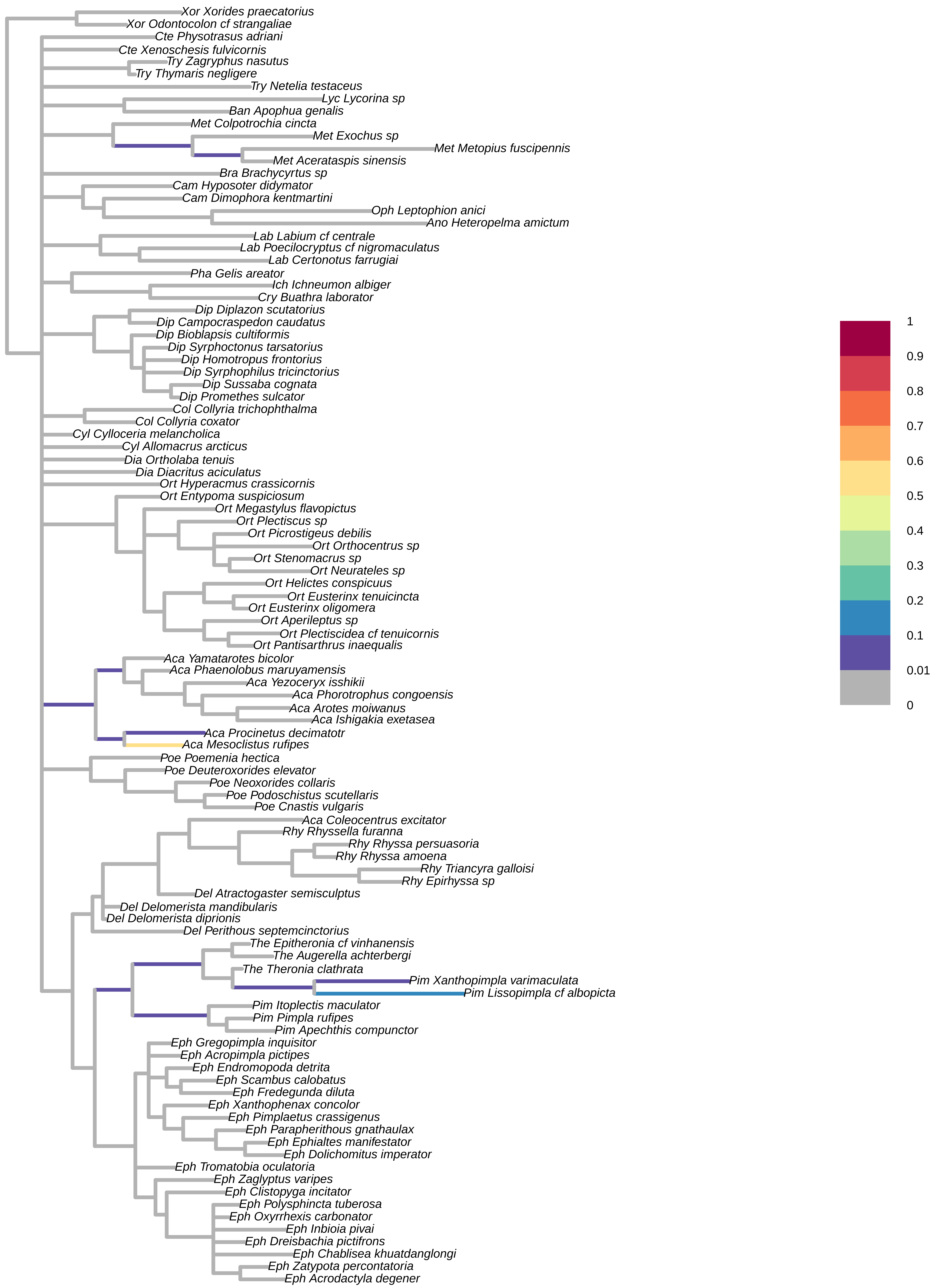

ICH_Mesornatus_markovici

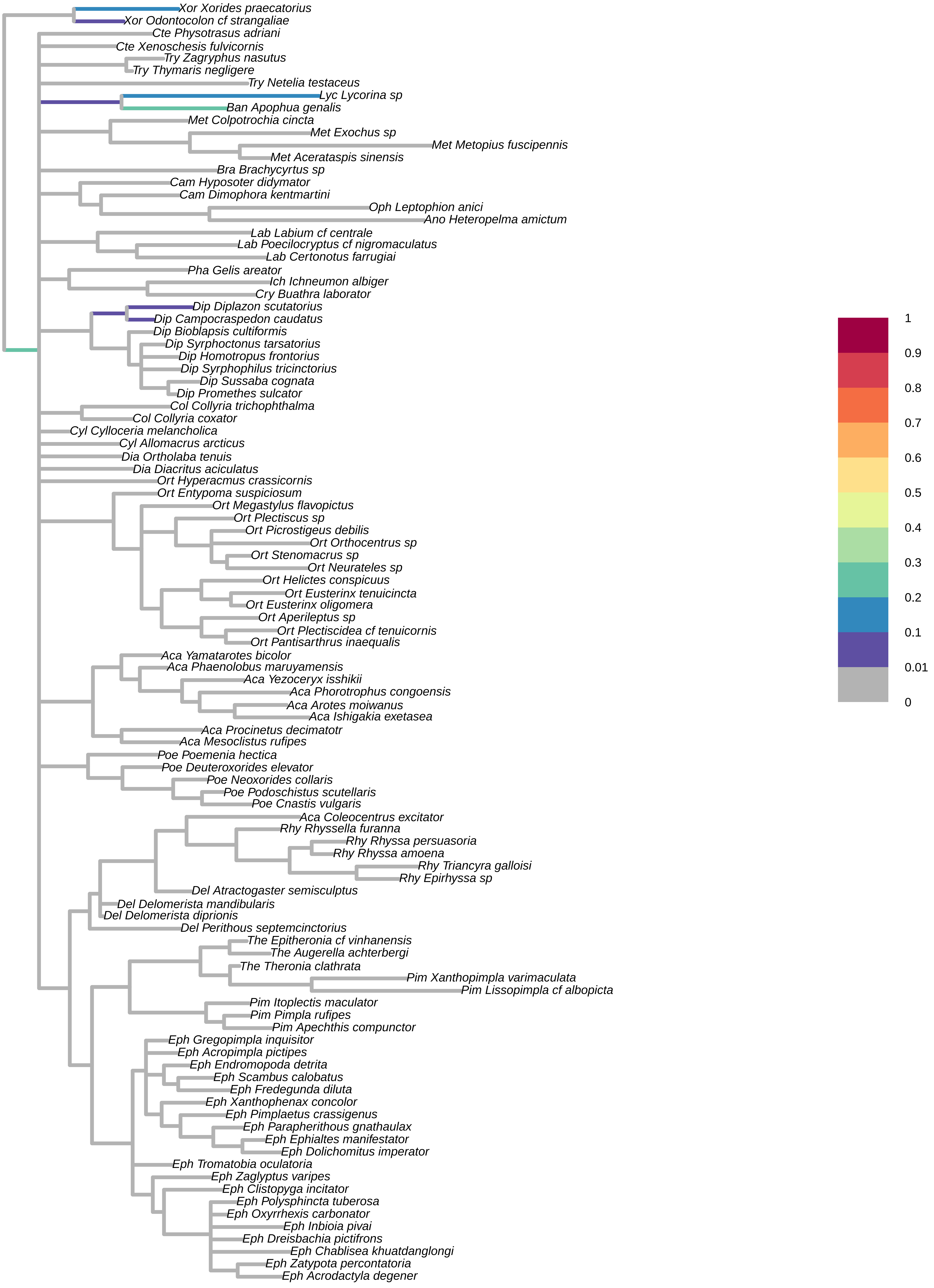

Eph_Scambus_parachuti

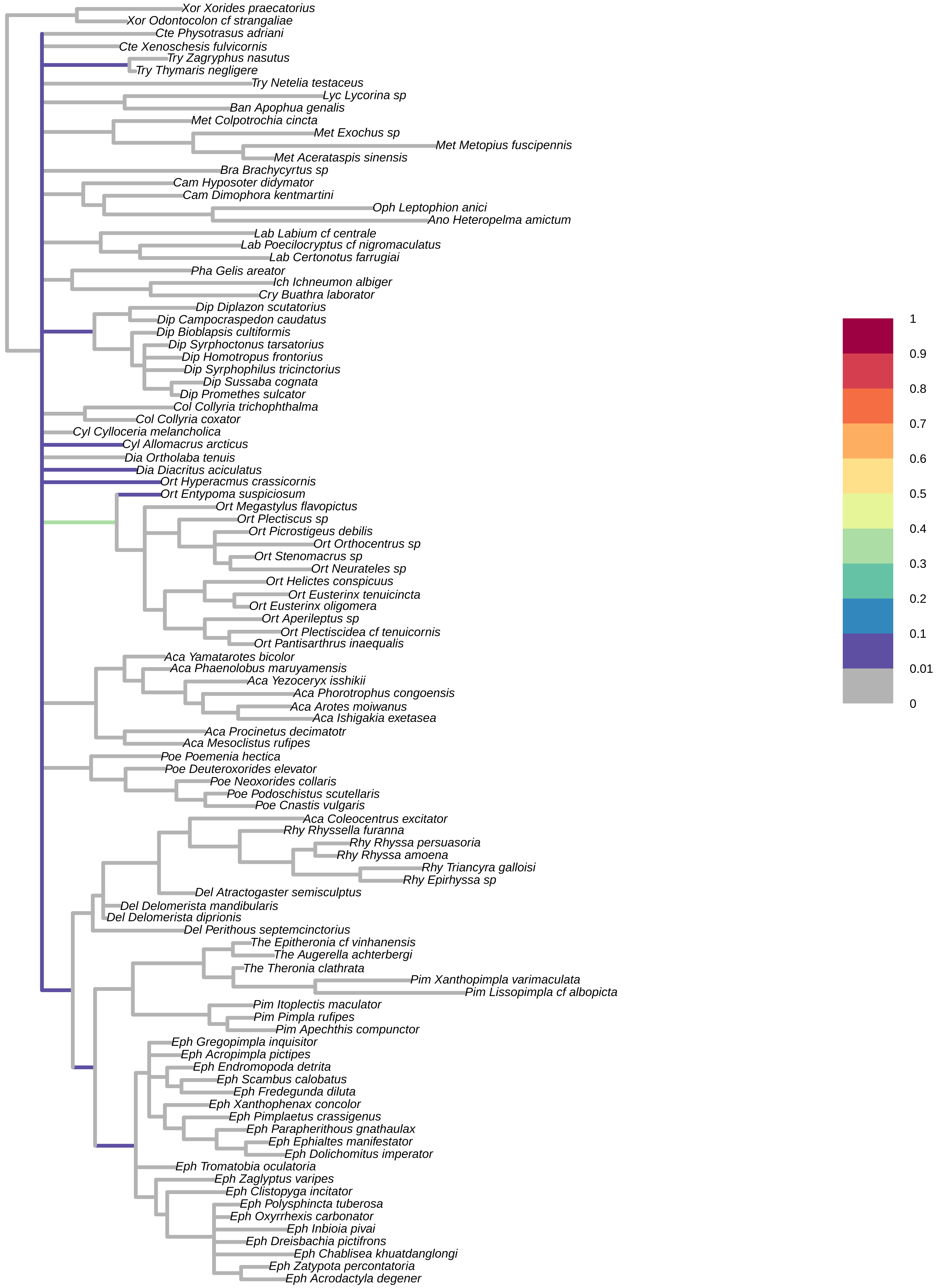

Lab_Trigonator_macrocheirus

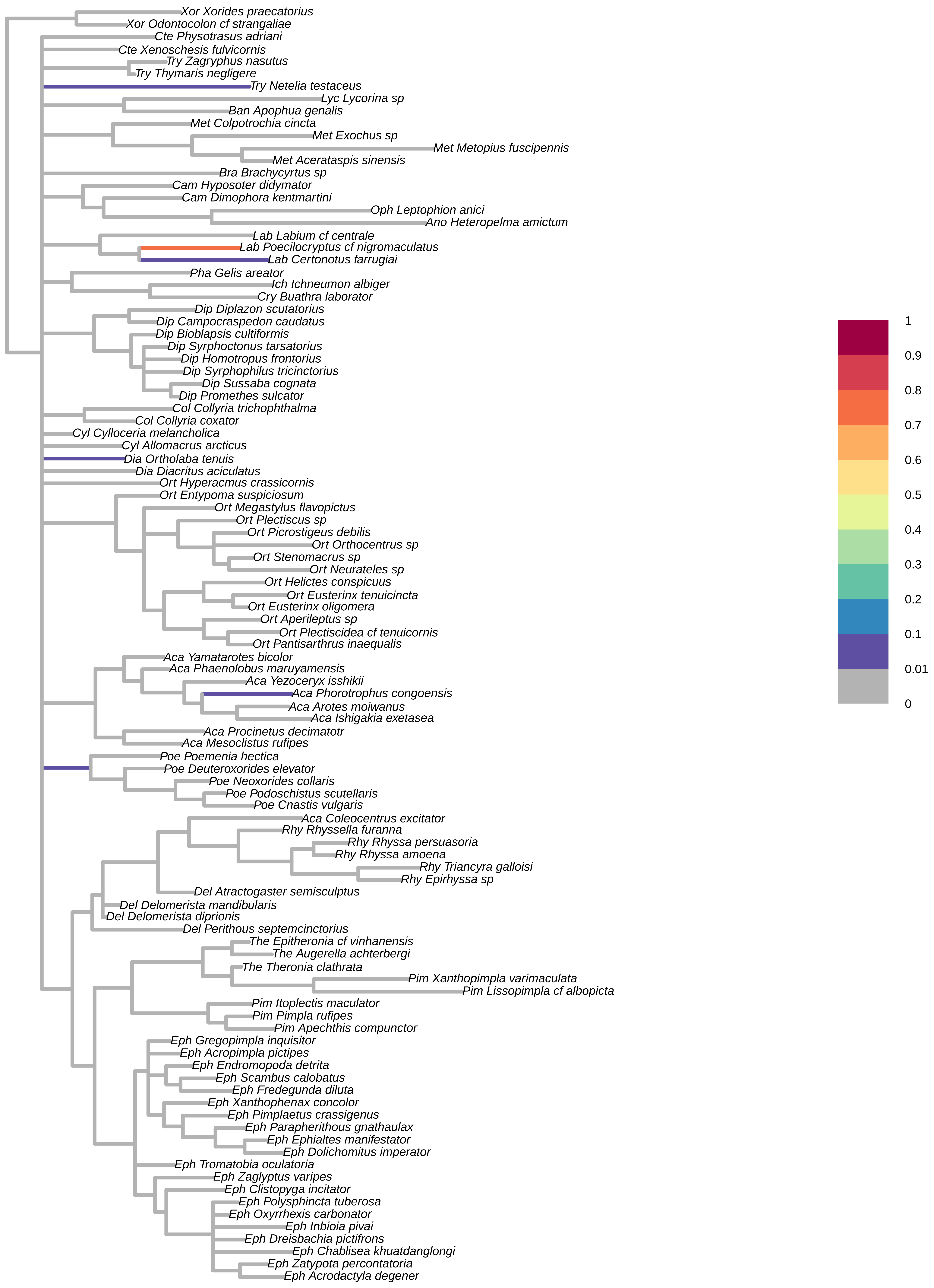

PIM_Xanthopimpla_praeclara

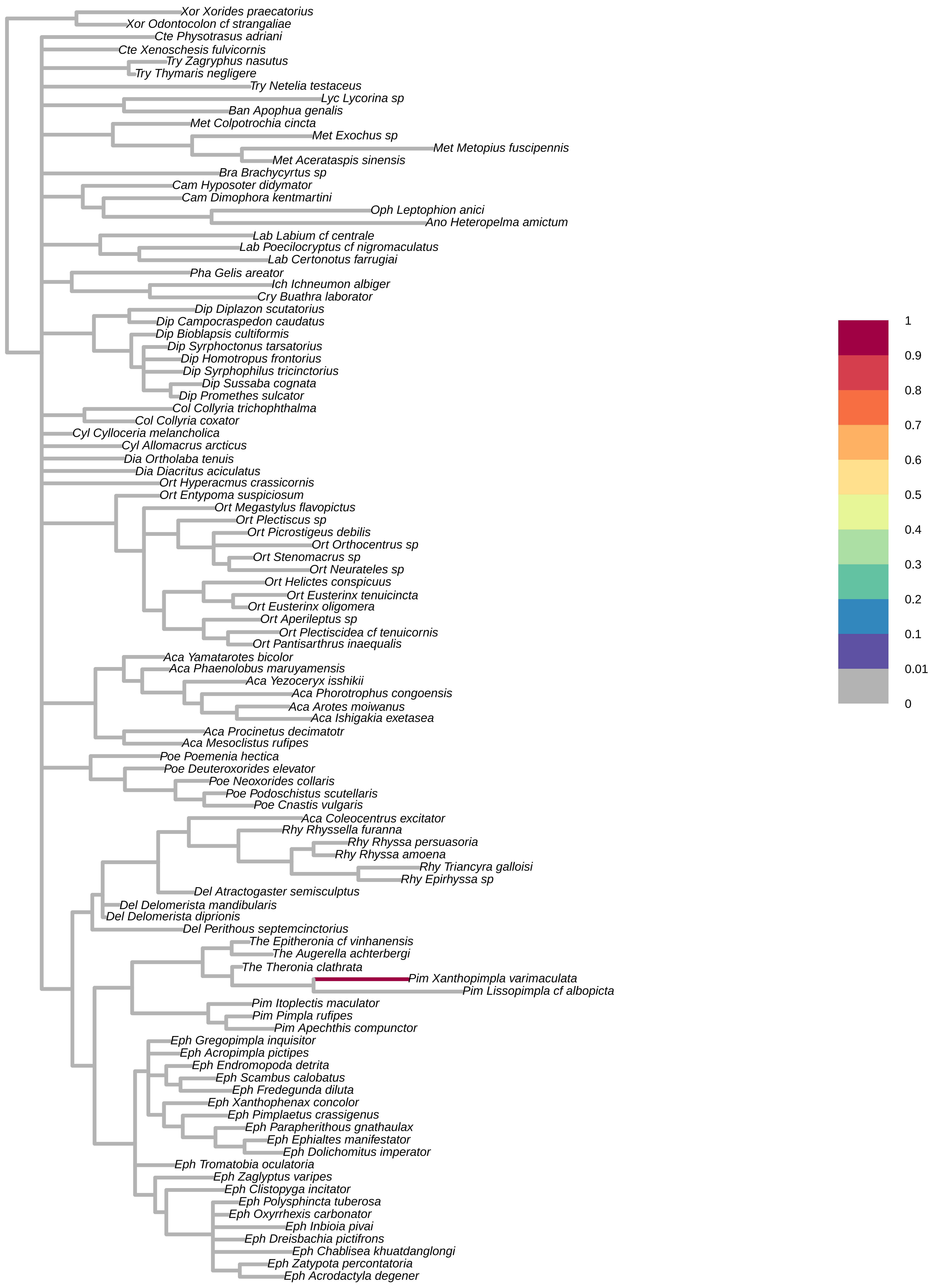

ICH_Polyhelictes_bipolarus

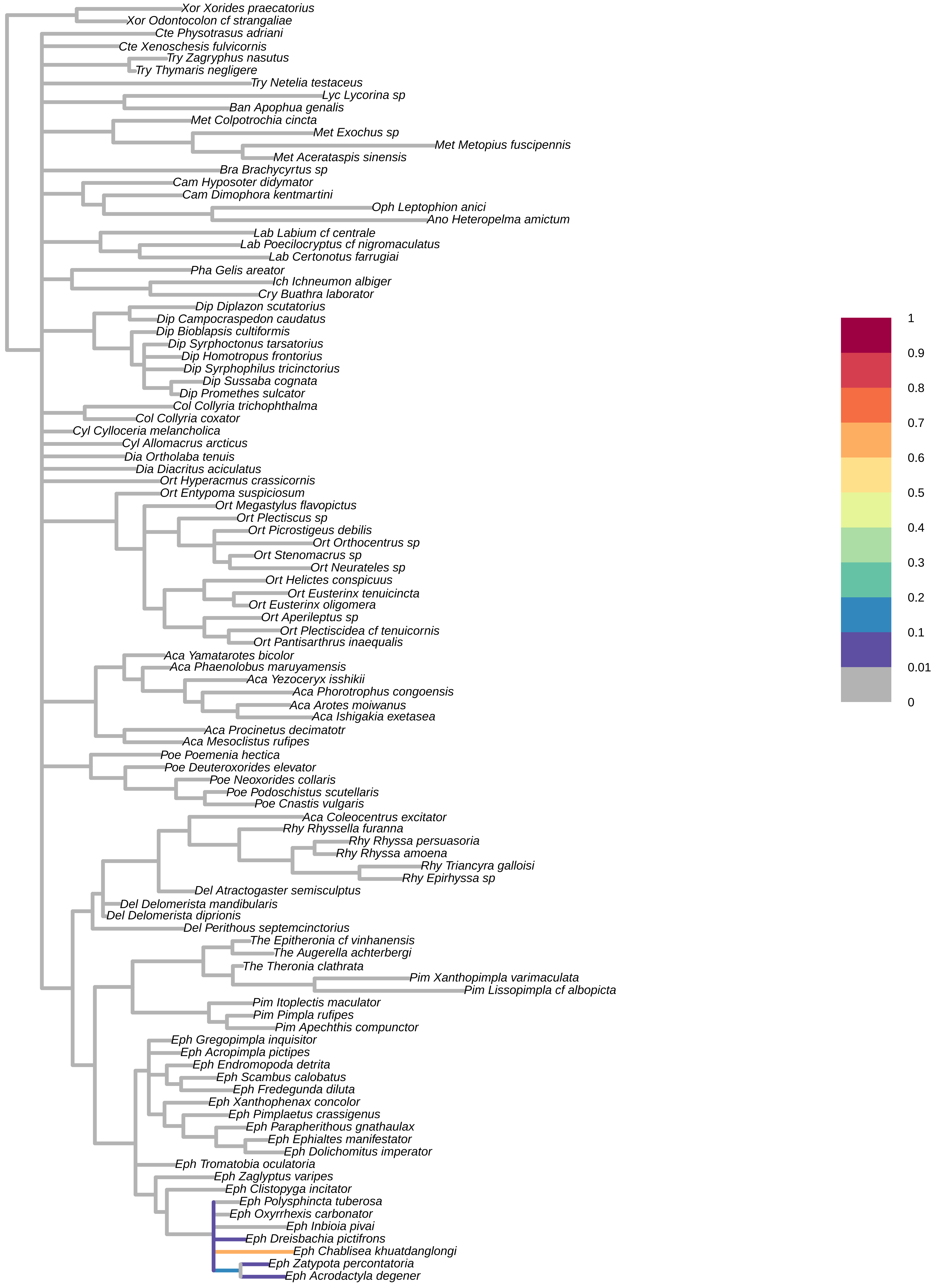

Eph_Scambus_fossilobus

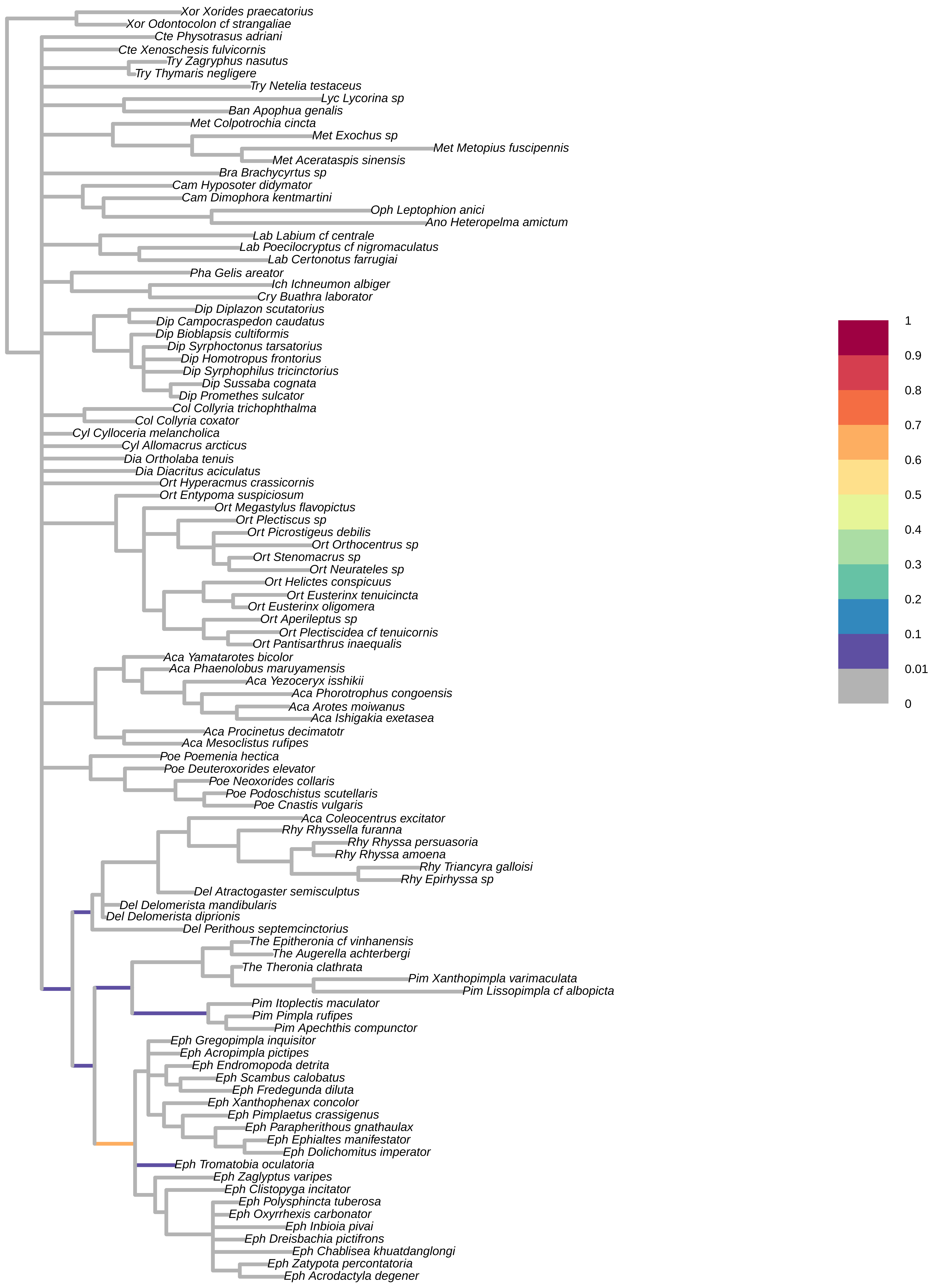

Rhy_Rhyssella_vera

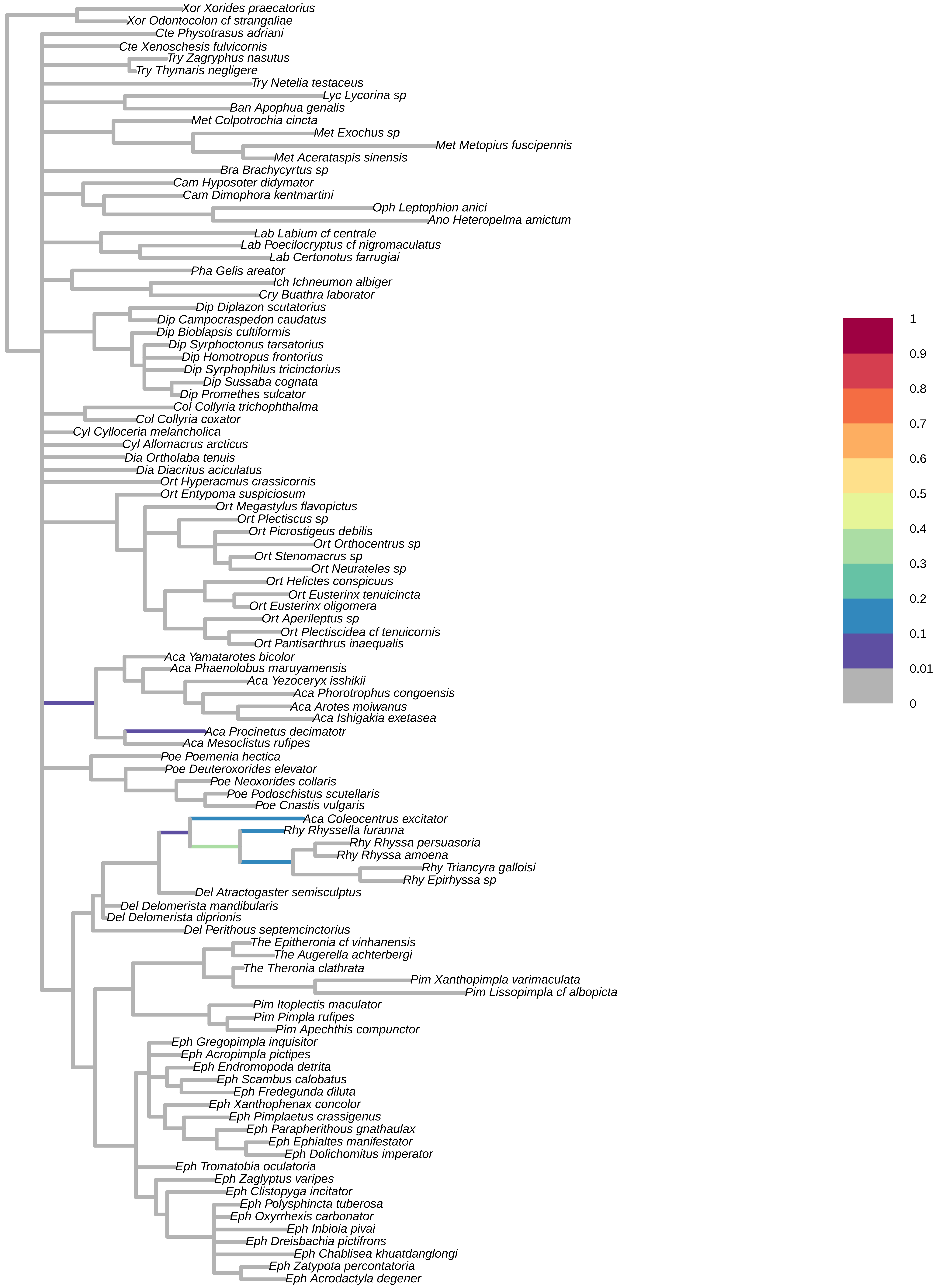

Eph_Scambus_mandibularis

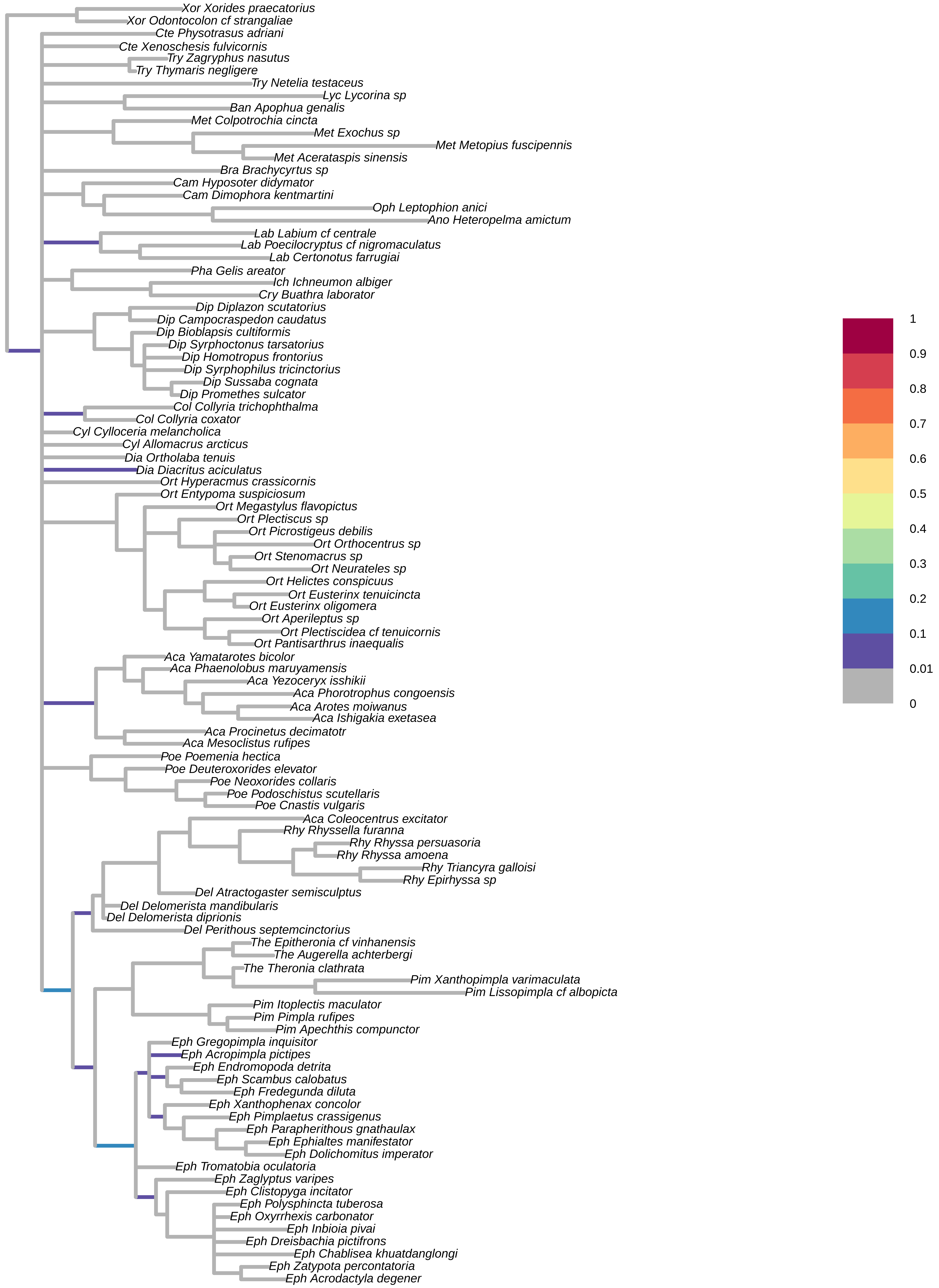

Supplement: S4 File — (PDF) [file pone.0212942.s005.pdf]
